# The genuinely multipartite nonlocality of graph states is model-dependent

**DOI:** 10.1038/s41534-025-01024-x

**Published:** 2025-06-02

**Authors:** Xavier Coiteux-Roy, Owidiusz Makuta, Fionnuala Curran, Remigiusz Augusiak, Marc-Olivier Renou

**Affiliations:** 1 Department of Computer Science, University of Calgary, Calgary, Canada; 2 Department of Physics and Astronomy, University of Calgary, Calgary, Canada; 3 Instituut-Lorentz, Universiteit Leiden, RA Leiden, The Netherlands; 4 Applied Quantum Algorithms Leiden, Leiden, The Netherlands; 5 Center for Theoretical Physics, Polish Academy of Sciences, Warsaw, Poland; 6 ICFO-Institut de Ciencies Fotoniques, The Barcelona Institute of Science and Technology, Castelldefels, Barcelona Spain; 7 Inria Saclay, Bâtiment Alan Turing, Palaiseau, France; 8 CPHT, Ecole polytechnique, Institut Polytechnique de Paris, Palaiseau, France

**Keywords:** Quantum information, Qubits

## Abstract

The notion of genuinely multipartite nonlocality (GMNL) was introduced to conceptualize the fact that nonclassical resources involving more than two parties in a nontrivial way may be needed to account for some quantum correlations. In this letter, we first recall the contradictions inherent to the historical definition of GMNL. Second, we turn to one of its redefinitions, called Local-Operations-and-Shared-Randomness GMNL (LOSR-GMNL), proving that all caterpillar graph states (including linear cluster states) have this second property. Finally, we conceptualize a third, alternative definition, which we call Local-Operations-and-Neighbour-Communication GMNL (LONC-GMNL), that is adapted to situations in which short-range communication between some parties might occur. We show that linear cluster states do not have this third property, while GHZ states do. Beyond its technical content, our letter illustrates that rigorous conceptual work is needed before applying the concepts of multipartite nonlocality or entanglement to benchmark the nonclassicality of some experimentally-produced quantum systems.

## Introduction

Bell’s 1964 theorem^1^ proved that two space-like separated parties can produce nonlocal correlations by performing local measurements on an appropriately entangled quantum system. This discovery of the nonlocality of quantum correlations has had a profound and lasting impact. At a fundamental level, quantum nonlocal correlations resist explanations in terms of local-hidden-variable models: they can score higher at Bell games (e.g., the CHSH game) than classical correlations^2–4^. In terms of concrete applications, Bell nonlocality allows for the certification of the properties or functionalities of quantum devices (e.g., entanglement of a state^5–7^ or a measurement^8,9^, security of a quantum key distribution protocol^10–12^) based solely on the objective operational correlations between experimental events.

In this *device-independent* (DI) paradigm^13^, the certification is obtained without making any assumptions on the measurement apparatus. Here, one does not even make any assumptions on the physical theory governing them (only assuming it satisfies causality^14^), allowing one to consider and test alternative descriptions of nature. As a result, DI is seen as the most secure way to assess the progress of quantum technologies, e.g., to benchmark a quantum device without needing to trust its designer or the measurement apparatus used to characterize it. This contrasts with the *device-dependent* (DD) paradigm, where quantum theory is accepted (e.g., Tsirelson’s $2\sqrt{2}$ bound for the CHSH game holds, while a score of up to 4 is possible in the DI paradigm^15^) and the detectors are trusted. Intermediary concepts of semi-device-independence have also been introduced^16,17^.

The ability to manipulate large nonclassical quantum systems is seen as a key resource in many applications of quantum theory. The desire to certify this ability in a DI way has motivated the concept of *nonlocality of depth n* (which we abbreviate as GMNL_*n*_). Systems that are GMNL_*n*_ are systems that produce correlations whose nonlocality cannot be understood as being ‘obtained by combining many states composed of at most (*n* − 1)-partite constituents’. Furthermore, when a system of *n* parties exhibits GMNL_*n*_ correlations, we say the system is (maximally) *genuinely multipartite nonlocal* (GMNL). A typical example of a state that should intuitively produce GMNL_3_ (hence GMNL) correlations is the three-party $\left\vert {{\rm{GHZ}}}_{3}\right\rangle =(\left\vert 000\right\rangle +\left\vert 111\right\rangle )/\sqrt{2}$ state, while, by contrast, the state $\left\vert {\phi }^{+}\right\rangle \otimes \left\vert 0\right\rangle$, which is composed of an EPR pair and an (independent) ancillary qubit, should intuitively produce only GMNL_2_ correlations.

**Fig. 1:**
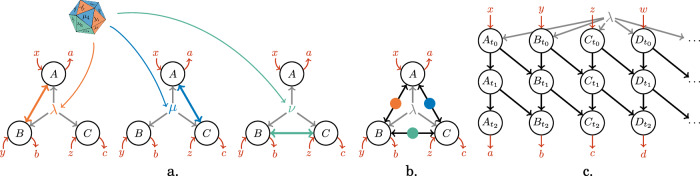
Three different formulations of the notion of genuine multipartite nonlocality. **a** Svetlichny’s LOCC causal explanatory model defines as GMNL the correlations $\overrightarrow{P}=\{p(a,b,c| x,y,z)\}$ that cannot be obtained in a causal structure in which one stochastically chosen party (selected by the die) is independent of the other two parties (e.g., if the die rolls *μ*_4_, then *B* is independent from *A*, *C*). Svetlichny’s GMNL correlations can thus be written as $\overrightarrow{P}\ne \int{\rm{d}}\lambda \,{\overrightarrow{Q}}_{AB}^{\lambda }{\overrightarrow{Q}}_{C}^{\lambda }+\int{\rm{d}}\mu \,{\overrightarrow{R}}_{BC}^{\mu }{\overrightarrow{R}}_{A}^{\mu }+\int{\rm{d}}\nu \,{\overrightarrow{S}}_{AC}^{\nu }{\overrightarrow{S}}_{B}^{\nu }$, where e.g., ${\overrightarrow{Q}}_{AB}^{\lambda }={\{{q}^{\lambda }(a,b| x,y)\}}_{\lambda }$ may be a signalling distribution. **b** In the LOSR redefinition^27^, $\overrightarrow{P}=\{p(a,b,c| x,y,z)\}$ is GMNL if it is not compatible with the represented causal model, where the principles of causality (also called No-Signalling and Independence) and of device-replication are assumed (see the proof of Theorem 1 and Fig. 3). Here, the shared randomness *λ* is tripartite, and the three bipartite sources are arbitrary no-signalling resources. **c** In this letter, we introduce the Local-Operations-and-Neighbour-Communication (LONC) framework, in which we define the LONC-GMNL correlations for a given underlying graph. Here we illustrate the correlations $\overrightarrow{P}=\{p(a,b,c,d,\ldots | x,y,z,w,\ldots \,)\}$ that can be generated in two rounds of one-way synchronous communication on a path (i.e., the correlations are LONC-GMNL_2_ for the directed path).

The task of extending this preliminary idea into a general classification of all states, that is, proposing a universal mathematical definition for the concept of GMNL, is not trivial. It is, however, of critical importance, as many experimental works assess the large nonclassicality of their experimentally-produced systems based on the notion of GMNL^18–21^. An appropriate definition requires first the introduction of a concrete physical model expressing what is meant by ‘obtained by combining many states composed of at most (*n* − 1)-partite constituents,’ which we call the *causal explanatory model*. Then, by definition, a state producing correlations that cannot be explained in this causal explanatory model will be called GMNL_*n*_. The first historical definition of GMNL was proposed by Svetlichny^22^ (see Fig. 1a for a detailed description) in the context of Local Operations and Classical Communication (LOCC). It characterizes GMNL as the direct DI counterpart to the DD definition by Seevinck and Uffink of *genuine multipartite entanglement*^23^. As we detail later, the LOCC definition by Svetlichny, as well as the one by Seevinck and Uffink, are known (since at least a decade ago^24^) to be paradoxical when used to assess the large nonclassical nature of practical quantum systems. This has motivated the redefinition of these concepts in the framework of Local-Operations-and-Shared-Randomness (LOSR), which resulted in a total redefinition of the underlying causal explanatory model to be excluded^25–27^, inspired by the concept of quantum network nonlocality^28–34^. In short, a probability distribution is said to be LOSR-GMNL if it **cannot** be reproduced by an LOSR network (see Fig. 1b. for the graphical representation and a detailed description of the underlying assumptions).

This type of redefinition leads naturally to the exclusion of some correlations that previously were GMNL (see e.g ref. ^26,35^). Therefore, it is important to characterize the correlations that admit both definitions or admit only one but not the other. Yet, very few states are known to produce GMNL correlations under the LOSR framework. In refs. ^25,26^ and ^27^ it was proven that $\left\vert {\rm{GHZ}}\right\rangle$ is GME and GMNL according to the redefinitions of Fig. 1b, which we denote by LOSR-GME and LOSR-GMNL respectively. The existence of LOSR-GMNL correlations has also been demonstrated experimentally^36,37^. Recent results have shown that *n*-partite graph states, a well-studied family of multipartite quantum states^38^ which we recall below, are LOSR-GME_3_^39,40^. Importantly, while appropriate in some situations, the new LOSR definition of Fig. 1b may not be suitable for all experiments. One could consider, for example, a 1*D* condensed matter system, in which subsystems are so close to each other that short-range communication between adjacent sites cannot be ruled out. In such a scenario, an alternative causal explanatory model should be used which allows for communication between neighbours. Allowing such short-range communication is also directly relevant to quantum distributed computing^41–44^, in which geographical latency limits but does not totally prohibit communication.

**Fig. 2:**
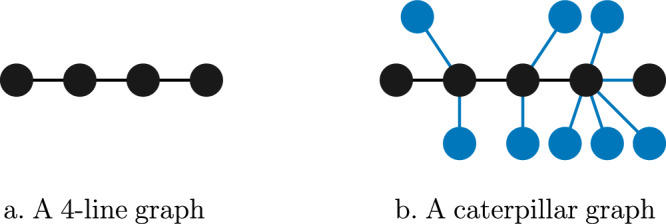
Graph states are defined from graphs. **a** A linear *n*-cluster state corresponds to a line over *n* vertices. **b** A caterpillar graph is a connected acyclic graph whose vertices of degree greater than one form a path (the “spine” in black above). All vertices of degree 1 (the “legs”, in blue above) hence connect only to the spine.

In this letter, we focus our analysis on the (more demanding) DI paradigm, discussing the implications for the (weaker) DD paradigm when applicable. We show that all linear cluster states, and in fact, all *caterpillar graph states* (shown in Fig. 2), are LOSR-GMNL, in a noise-robust way. Our results improve significantly on previous techniques, which could only provide lower bounds of degree 3 on the nonlocality of those states, and were restricted to the weaker device-dependent setting of GME^39,40^. This is thus the main technical contribution of our letter. Second, motivated by experimental considerations and distributed computing, we introduce a new framework allowing Local Operations and Neighbour Communication (LONC). Its associated causal explanatory model (see Fig. 1c) follows *t* communication steps along a given network. This leads us to a new notion of certifiable multipartite nonlocality which we call LONC-GMNL, according to which states can be classified quantitatively as LONC-GMNL_*t*_ for a given network. We prove that, in this new LONC-GMNL definition, $\left\vert {\rm{GHZ}}\right\rangle$ and linear cluster states belong respectively to opposite ends of the complexity spectrum: for the directed path communication graph, $\left\vert {\rm{GHZ}}\right\rangle$ is maximally LONC-GMNL while the linear cluster state is only LONC-GMNL_2_ (this finding improves a result of^44^). Characterizing this distinction by introducing the LONC framework is the main conceptual contribution of our letter.

## Results

### Preliminaries

#### LOCC and LOSR lead to two different notions of GMNL

The historical Svetlichny definition of GMNL is anchored in the LOCC framework and is based on the explanatory model of Fig. 1a. There are two important issues with this model. First, multiple copies of few-partite states distributed among different parties can simulate many-partite GMNL. For example, the composition ${\left\vert {\phi }^{+}\right\rangle }_{A{B}_{1}}\otimes {\left\vert {\phi }^{+}\right\rangle }_{{B}_{2}C}$ of two bipartite EPR states, where *A*, *C* respectively own one qubit and *B* owns two qubits, is Svetlichny-GMNL_3_ despite comprising only bipartite sources (more generally, *n* copies of $\left\vert {\phi }^{+}\right\rangle$ can be Svetlichny-GMNL_*n*+1_). This is highly problematic in a device-independent context where the large nonclassicallity of a quantum source should be benchmarked without trusting its designer or the measurement apparatus. Indeed, a designer only able to produce bipartite $\left\vert {\phi }^{+}\right\rangle$ states could claim to produce GMNL_*n*_ states with arbitrarily large *n* (Note that while the bipartite structure of ${\left\vert {\phi }^{+}\right\rangle }_{A{B}_{1}}\otimes {\left\vert {\phi }^{+}\right\rangle }_{{B}_{2}C}$ is apparent, *B* could obfuscate that structure by encoding their state in a four-level system and applying some local unitary.). Second, more conceptually, this definition involves signaling distributions (see caption of Fig. 1a), which is not physically motivated. While the latter shortcoming was addressed in^35^ and the modified definition has been studied in subsequent works (see e.g., ref. ^45^), patching the first issue required a shift of the concept of GMNL from the LOCC framework towards the LOSR framework. This led to the introduction of the causal explanatory model which we detail in Fig. 1b.

The LOSR and LOCC have been studied a lot from the perspective of state preparation and the resource theories of entanglement and nonlocality^46–52^. However, in this paper we take a different approach— we do not study the correlations or states arising from these models; rather, we wish to disprove them as the origin of a particular correlation.

#### Graph states and linear cluster states

Graph states are a family of states corresponding to graphs where vertices represent subsystems and edges determine the entanglement between these subsystems (see Fig. 2). Due to their inherent symmetries, as well as due to the simplicity of applying Clifford gates on graph states, these states have found numerous applications in quantum computation^53–56^, quantum error correction^57,58^, quantum cryptography^59,60^, and even quantum metrology^61,62^. Furthermore, their high degree of nonclassicality has made them a frequent subject of entanglement^38,63^ and nonlocality^64–66^ studies.

In our main text, we focus for simplicity on the linear 4-cluster state $\left\vert {C}_{4}\right\rangle$, which is a graph state corresponding to four vertices on a line. For a particular choice of local basis, it can be written as

1 $$\left\vert {C}_{4}\right\rangle := \frac{1}{\sqrt{2}}\left(\left\vert 00\right\rangle \left\vert {\phi }^{+}\right\rangle +\left\vert 11\right\rangle \left\vert {\phi }^{-}\right\rangle \right)\,,$$

where $\left\vert {\phi }^{\pm }\right\rangle := \frac{1}{\sqrt{2}}\left(\left\vert 00\right\rangle \pm \left\vert 11\right\rangle \right)$. For a more detailed introduction to graph states, see Supplementary Material I.

### Main results

#### All caterpillar graph states are genuinely multipartite nonlocal

This section contains the main technical contribution of our letter, which is to prove that all caterpillar graph states are LOSR-GMNL in a noise-tolerant way. Here, we present the proof idea (for the detailed proof, see Supplementary Material II) using as an example the 4-partite linear cluster state $\left\vert {C}_{4}\right\rangle$, which is one of the simplest representatives of caterpillar graph states. The general proof for any caterpillar graph state is relegated to Supplementary Material III.

##### Theorem 1

All caterpillar graph states are LOSR-GMNL in a noise-robust way.

In particular, any state violating the following is GMNL_4_,

2 $$2\langle {A}_{0}{B}_{0}\rangle +2\langle {C}_{0}{D}_{2}\rangle +2\langle {A}_{1}{B}_{1}{D}_{2}\rangle +{I}^{BCD}\leqslant 8\,,$$

where *I*^*BCD*^ ≔ 〈*C*_0_*D*_0_〉 + 〈*C*_0_*D*_1_〉 + 〈*B*_0_*C*_1_*D*_0_〉 − 〈*B*_0_*C*_1_*D*_1_〉 is a distributed variant of the CHSH inequality.

Under suitable measurements, the $\left\vert {C}_{4}\right\rangle$ linear cluster state violates this bound by reaching $6+2\sqrt{2}\, > \,8$.

In the above, by noise-robust we mean that for any *N*-partite caterpillar graph state $\left\vert {\ddagger }_{N}\right\rangle$, there exists a threshold value *η*_thr_ < 1 such that for all *η* > *η*_thr_, the state

3 $${\rho }_{{\ddagger }_{N}}(\eta )=\eta \left\vert {\ddagger }_{N}\right\rangle \left\langle {\ddagger }_{N}\right\vert +\frac{1-\eta }{{2}^{N}}{\mathbb{1}}\,$$

is LOSR-GMNL.

##### Proof

The proof of Eq. (2) is based on the inflation technique^27,67,68^. We assume that in the underlying causal model, the alternative explanation for the observed correlations is compatible with the principle of causality^14^, also called No-Signalling and Independence^69,70^.

**Fig. 3:**
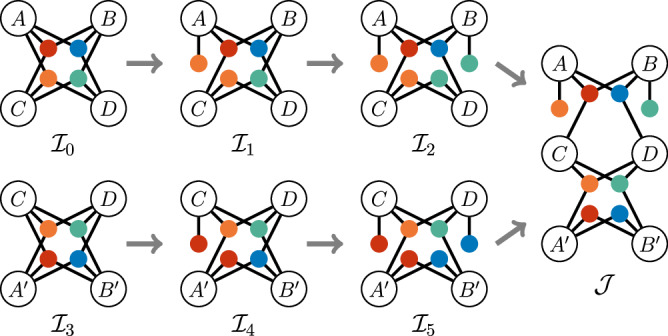
Visual representation of the inflation used in the proof of Ineq. (2) in Theorem 1. The colored disks represent general tripartite nonsignalling sources and the lettered disks are the parties. For clarity, we do not include in the figure classical randomness shared by all parties.

Here we present a simplified version of the proof in which we show that the LHS of Ineq. (2) cannot reach the value of $6+2\sqrt{2}$ if the underlying correlations originate from a network ${{\mathcal{I}}}_{0}$ (see Fig. 3). To this end, we make use of a proof by contradiction - we first assume that the quantum violation $\langle {A}_{0}{B}_{0}\rangle =\langle {C}_{0}{D}_{2}\rangle =\langle {A}_{1}{B}_{1}{D}_{2}\rangle =1,\,{I}^{BCD}=2\sqrt{2}$ can be obtained without any 4-way nonclassical cause, that is, it can be obtained from four 3-way nonclassical causes as in the networks ${{\mathcal{I}}}_{0},{{\mathcal{I}}}_{3}$ presented on Fig. 3.

We show that if our assumption held true, it would imply that in the rightmost inflated scenario ${\mathcal{J}}$ on Fig. 3, *C* would be part of the violation of a distributed variant of the CHSH inequality ${I}_{({\mathcal{J}})}^{ACD}$ (the quantity *I*^*ACD*^ is defined as *I*^*BCD*^ but with Bob replacing Alice), but his output could be guessed by ${A}^{{\prime} },{B}^{{\prime} }$ together, which is impossible as it contradicts the monogamy of CHSH correlations.

We reach this conclusion by following two different lines of inflation reasonings: ${{\mathcal{I}}}_{0}\to {{\mathcal{I}}}_{1}\to {{\mathcal{I}}}_{2}\to {\mathcal{J}}$ (top path on Fig. 3) and ${{\mathcal{I}}}_{3}\to {{\mathcal{I}}}_{4}\to {{\mathcal{I}}}_{5}\to {\mathcal{J}}$ (bottom path on Fig. 3). We repeatedly use the argument that the behaviour of two subgroups of parties in two of these networks ${{\mathcal{I}}}_{k},{{\mathcal{I}}}_{l}$ must be identical whenever their associated subnetworks are isomorphic. For instance, isolate *B*, *C* and *D* in ${{\mathcal{I}}}_{0}$ and ${{\mathcal{I}}}_{1}$. As their associated subnetworks (that is, the subnetworks in ${{\mathcal{I}}}_{0},{{\mathcal{I}}}_{1}$ that are composed of *B*, *C*, *D* and the sources they are connected to) are isomorphic, we must have ${I}_{({{\mathcal{I}}}_{0})}^{BCD}={I}_{({{\mathcal{I}}}_{1})}^{BCD}$. Since we assumed that ${I}_{({{\mathcal{I}}}_{0})}^{BCD}=2\sqrt{2}$, we deduce that ${I}_{({{\mathcal{I}}}_{1})}^{BCD}=2\sqrt{2}$. By repeatedly using this type of argument along these networks, it is possible to show that $(i):{\langle {A}_{1}^{{\prime} }{B}_{1}^{{\prime} }{C}_{0}\rangle }_{({\mathcal{J}})}=1$ and $(ii):{I}_{({\mathcal{J}})}^{ACD}=2\sqrt{2}$. (*i*) implies that the product of the outputs of ${A}^{{\prime} },{B}^{{\prime} }$ on their input 1 is equal to the output of *C* on his input 0. (*ii*) shows that the output of *C* on input 0 is part of a violation of the distributed variant of the CHSH inequality ${I}_{({\mathcal{J}})}^{ACD}\le 2$. This directly contradicts the monogamy of nonlocal correlations, which states that outputs violating CHSH cannot be guessed with high probability by a third party^71^. Therefore, we have shown that the correlations originating from ${{\mathcal{I}}}_{0}$ cannot achieve the value of $6+2\sqrt{2}$.

However, quantum correlations can achieve this value, which can be easily shown by measuring on the state ${\left\vert {C}_{4}\right\rangle }_{ABCD}$ the following observables: *Z* for *A*_0_, *B*_0_, *C*_0_, *D*_2_; *X* for *A*_1_, *B*_1_, *C*_1_; and, respectively, $(Z+X)/\sqrt{2},(Z-X)/\sqrt{2}$ for *D*_0_, *D*_1_. □

#### Higher dimensions

We show in Supplementary Material IV that linear cluster graph states of higher local dimensions (beyond qubits) are LOSR-GMNL for any prime local dimension and any number of subsystems, and in Supplementary Material V we show that the GHZ state is LOSR-GMNL for any local dimension and any number of subsystems.

#### From LOSR to LONC

We now turn to the main conceptual contribution of our letter, which is to adapt the concept of GMNL to the Local-Operations-and-Neighbour-Communication (LONC) framework, which relaxes the no-signalling assumption of the LOSR framework. We recall that the concept of LOSR-GMNL, shown in Fig. 1b, relies on the assumption that absolutely no communication occurs between any of the parties. This can be overly restrictive in experimental setups with a high number of parties in close proximity. For instance, in a 1*D* condensed matter system, one could imagine that each site cannot be realistically prohibited from exchanging information carriers with its neighbours during the time window separating the choice of the measurement setting (i.e. the input) and the reading of the measurement outcome (i.e. the output). As we explain below, even though linear cluster states are LOSR-GMNL when no communication is allowed (see Theorem 1), they can be prepared in only a few rounds of communication between neighbouring sites, and this communication can even be synchronous.

The above considerations motivate the introduction of the concept of Neighbour-Communication Genuine Multipartite Nonlocality (LONC-GMNL), which is inspired by synchronous distributed computing^41–44^. As detailed in Fig. 1c, our LONC model is based on a communication graph along which the parties, represented by nodes, can communicate with their neighbours during *t* synchronous communication steps (of unbounded bandwidth), and have access to shared randomness. Parties can transmit classical, quantum, or, more generally, any *causal resource*: the communication is only restricted by constraints of the no-signalling type^68,70^. The edges of the graph, which can be oriented or not, indicate the communication flow. Importantly, the parties receive their measurement inputs *before* the communication rounds, so inputs can be leaked to neighbours residing up to a distance *t* away.

Hence, the notion of LONC-GMNL is always relative to a specific communication graph, based on which the notion of neighbour communication is defined. This graph should, in practice, be selected according to the experiment topology. For simplicity, in the following, we focus on the 1*D*-local scenario, where all parties form a line and where communication flows in one direction only.

#### Linear cluster states are only LONC-GMNL_2_; but GHZ states are maximally LONC-GMNL

We show in Supplementary Material VI that two rounds of one-way quantum communication on a directed path are enough to *prepare* a linear cluster state $\left\vert {C}_{n}\right\rangle$:

##### Theorem 2

All linear cluster states $\left\vert {C}_{n}\right\rangle$ can be prepared in two rounds of one-way synchronous quantum communication.

This strengthens a result in^44^, where two-way communication was considered, and it shows that, while $\left\vert {C}_{n}\right\rangle$ is hard in the model of Fig. 1b, it is easy in that of Fig. 1c.

Note that Theorem 2 even allows one to *prepare*$\left\vert {C}_{n}\right\rangle$ with *quantum* communication. Hence, it only uses a very restricted part of what our LONC-GMNL model allows for: more generally, super-quantum communication can be used (as long as it does not allow for signalling), and it is sufficient to merely *simulate* the correlations (e.g., by leaking the input). For instance, it is evident that $\left\vert {{\rm{GHZ}}}_{n}\right\rangle$ cannot be constructed in the same way with less than *n* − 1 communication steps (because $\left\vert {{\rm{GHZ}}}_{n}\right\rangle$ is a coherent superposition of perfect correlations between all parties); it is, however, not straightforward to show that $\left\vert {{\rm{GHZ}}}_{n}\right\rangle$ is hard in the LONC-GMNL model. In particular, the leaking of inputs up to a distance *t* offers many ways to simulate some $\left\vert {{\rm{GHZ}}}_{n}\right\rangle$ correlations without necessarily constructing the state.

Despite these difficulties, we conclude this letter by showing that $\left\vert {{\rm{GHZ}}}_{n}\right\rangle$ is maximally LONC-GMNL for the oriented path graph.

##### Theorem 3

$\left\vert {{\rm{GHZ}}}_{n}\right\rangle$ produces correlations that are incompatible with the LONC model along an oriented path with *t* < *n* − 1 communication steps.

##### Proof

We prove this theorem in Supplementary Material VI, where we provide a Bell-like inequality that holds for correlations produced with *t* < *n* − 1 communication steps on the path *A*^(1)^ → ⋯ → *A*^(*n*)^, but that is violated by suitable measurements of the $\left\vert {{\rm{GHZ}}}_{n}\right\rangle$ state. □

Lastly, let us mention the relationship between this result and ref. ^72^. There, it was shown that correlations resulting from Pauli measurements on graph states can be simulated in some particular networks with one round of classical communication between neighbors, meaning that such correlations cannot be LONC-GMNL_1_. This may seem to contradict our result for $\left\vert {{\rm{GHZ}}}_{n}\right\rangle$ as this state is equivalent to a graph state up to local unitaries. This is not the case, however, since in our construction one party has to perform non-Pauli measurements.

## Discussion

In this letter, we presented three definitions of genuinely multipartite nonlocality, based on three different frameworks (i.e. LOCC, LOSR, and LONC) and on which causal explanatory model is to be rejected (see Fig. 1). After discussing the contradictions inherent to the standard Svetlichny LOCC definition (Fig. 1a), we turned to the model of Fig. 1b and showed in a noise-robust way that in this LOSR definition, caterpillar graph states are LOSR-GMNL. We then proposed the new LONC model of Fig. 1c as an intermediate model between LOSR and LOCC which allows communication between nearest neighbours. We showed that, in the directed path graph, linear cluster graph states are trivial while the $\left\vert {{\rm{GHZ}}}_{n}\right\rangle$ state is maximally LONC-GMNL.

While we only discussed the theory-agnostic DI setting (which does not limit the underlying causal explanatory model of Fig. 1 to quantum mechanics, and where, for example, nonlocal boxes could be transmitted), it is straightforward to adapt our results and concepts to the DD setting considered in^25,39,40^.

Let us conclude with a discussion of experimental benchmarking based on the concept of GMNL, often used to assess the large, nonclassical behavior of experimental systems^18–21^. As we have discussed, many different definitions of GMNL can be considered, leading to radically different conclusions. This begs the question, “*Which definition is the right one?*” We are convinced that no definitive answer can be given a priori. To select the most appropriate framework, one must first carefully reflect on the experimental setup under observation and on which causal explanatory model of the experimental observations should be ruled out. As we discussed, Svetlichny’s LOCC definition, designed to reject the causal explanatory model of Fig. 1a, is often ill-adapted as it fails to reject causal explanations based on bipartite sources. While an LOSR definition based on the causal explanatory model of Fig. 1b is appropriate under strict conditions of no-signalling, experimentalists working with many subsystems in close proximity might be more interested in refuting models allowing communication between nearest neighbours, as in the LONC causal explanatory model of Fig. 1c. However, one can also treat LONC-GMNL as a stronger certificate for multipartite nonlocality experiments, i.e., one can design experiments such that all of the parties measure simultaneously, and use LONC-GMNL as an extra layer of security. In that case, even if some measurements were slightly delayed with respect to others, if that delay is not too large, one can still argue for nonlocality detection based on the violation of LONC-based inequalities. Some other experimental situations might require the definition of a novel, custom model. Hence, before discussing the nature (or depth) of the genuine multipartite entanglement and genuinely multipartite nonlocality of a quantum system produced by some experiment, it is essential to do some rigorous conceptual work to identify a framework and definition that justify, in alignment with the experimental setup, which causal explanatory model is sensible.

## Supplementary information

Supplementary Information

## Data Availability

No datasets were generated or analysed during the current study.

## Code availability

No codes were generated or used in the current study.
