# Supplementary material for: The genuinely multipartite nonlocality of graph states is model-dependent
Source: npj Quantum Inf. 2025 Jun 2;11(1):90. doi: 10.1038/s41534-025-01024-x (PMC12129809; doi:10.1038/s41534-025-01024-x)
Supplement: Supplementary file 1 — Supplementary Information [file 41534_2025_1024_MOESM1_ESM.pdf]

Supplementary material: The genuinely multipartite nonlocality of graph states is model-dependent

Xavier Coiteux-Roy,[1,*] Owidiusz Makuta,[2,*] Fionnuala Curran,[3] Remigiusz Augusiak,[2] and Marc-Olivier Renou[4,5]

[1] *School of Computation, Information and Technology, Technical University of Munich & Munich Center for Quantum Science and Technology (MCQST), Munich, Germany.*

[2] *Center for Theoretical Physics, Polish Academy of Sciences, Aleja Lotników 32/46, 02-668 Warsaw, Poland.*

[3] *ICFO-Institut de Ciencies Fotoniques, The Barcelona Institute of Science and Technology, Castelldefels (Barcelona), Spain.*

[4] *Inria Saclay, Bâtiment Alan Turing, 1, rue Honoré d'Estienne d'Orves 91120 Palaiseau.*

[5] *CPHT, Ecole polytechnique, Institut Polytechnique de Paris, Route de Saclay – 91128 Palaiseau.*

I. INTRODUCTION TO GRAPH STATES

In this section, we give a comprehensive introduction to the graph states relevant from the perspective of our work. However, to properly define graph states, we first have to introduce the basic notions of graph theory. A graph G is defined as a pair of sets $G = (V, E)$ where V is a set of elements called vertices, while E is the set consisting of edges connecting elements from V. In this work, we make the most commonly used assumption that each individual edge connects exactly two, different vertices. However, in contrast to what is usually assumed, we consider graphs in which two vertices can be connected by more than one edge. Such graphs are commonly referred to as multigraphs, but here we do not make that distinction and refer to them simply as graphs.

Among the many properties of graphs that are commonly studied, three are of particular interest in the context of our work. The first one is connectivity: we say that a graph is connected if there exists a path from any vertex to any other vertex in the graph, i.e. we can get from any vertex to any other vertex by traveling along the edges of the graph.

The second definition of interest to us is that of an induced subgraph $G_S = (V_S, E_S)$ of a graph $G = (V, E)$, which is defined by the following relations:

$$V_S \subseteq V\,, \qquad E_S = \{\{i,j\} \in E | i,j \in V_S\}\,. \tag{1}$$

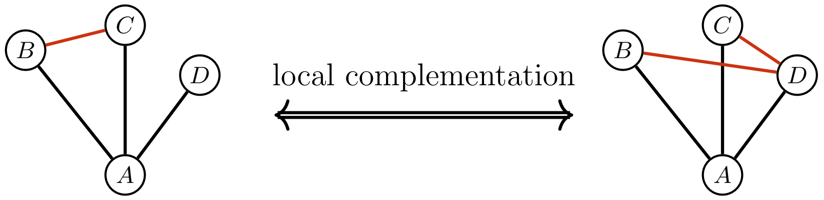

(a) Local complementation acting on A, for dimension $d = 2$.

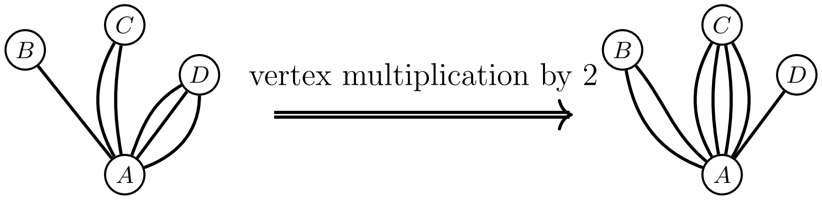

(b) Vertex multiplication by 2 acting on A, for dimension $d = 5$.

FIG. 1.

[*] These two authors contributed equally.

In descriptive terms, an induced subgraph G_S is constructed from the graph G by removing some vertices of G and all edges related to the removed vertices. The third important graph property is the neighbourhood $\mathcal{N}_i$ of a vertex i. It is defined as a set of all vertices in V connected to i by at least one edge

$$\mathcal{N}_i = \{j \in V | \{i,j\} \in E\}\,. \tag{2}$$

Using these three properties, we can define the classes of graphs on which we focus in our work. The first class, called linear graphs (or path graphs), is pretty self-explanatory: it is a graph that forms a line. In more technical terms, a graph $G = (V, E)$ is a linear graph if it is connected and if, for exactly two vertices $i, j \in V$, we have $|\mathcal{N}_i| = |\mathcal{N}_j| = 1$, while $|\mathcal{N}_k| = 2$ for all $k \in V \setminus \{i, j\}$. In order to define the second class, we first need to consider the induced subgraph $G_S = (V_S, E_S)$ of $G = (V, S)$ defined by

$$V_S = \{i \in V | |\mathcal{N}_i| \geqslant 2\}\,. \tag{3}$$

If G is connected and the induced subgraph G_S is a linear graph, then we call G a caterpillar graph. In other words, a caterpillar graph is a path graph with additional vertices attached to it, such that the only vertex in their neighbourhood is one of the vertices in the path graph. See Fig. 3 for an example of a caterpilar graph.

While the formal definition of a graph and its graphical description both prove useful for some applications, they are unsuitable for an efficient mathematical analysis of a graph structure. To that end, one considers an adjacency matrix Γ of a graph G. The matrix Γ is defined element-wise: a matrix element $\Gamma_{i,j}$ corresponds to the number of edges $\{i, j\} \in E$, connecting vertices $i, j \in V$, where we label vertices as $V = \{1, 2, \ldots, N\}$.

There are three properties of Γ worth highlighting. First, each element $\Gamma_{i,j}$ has to be a natural number (including zero), as vertices can be connected only by a discrete number of edges, but this number can be larger than one, as we consider multigraphs. Second, Γ has to be symmetric, as the number of edges connecting i and j is the same as the number of edges connecting j and i. Lastly, since we exclude edges that connect a vertex to itself, we have $\Gamma_{i,i} = 0$ for all $i \in V$.

With that, we are ready to define graph states. Let us consider a Hilbert space $\mathcal{H} = \mathbb{C}_d^{\otimes N}$ and generalised Pauli matrices

$$X = \sum_{j=0}^{d-1} |j+1\rangle\langle j|\;, \qquad Z = \sum_{j=0}^{d-1} \omega^j\, |j\rangle\langle j|\;, \tag{4}$$

where $\omega = \exp(2\pi\mathbb{i}/d)$ and $|d\rangle \equiv |0\rangle$. Given $\mathcal{H}$ and a graph G, a graph state $|G\rangle \in \mathcal{H}$ is defined as the unique state fulfilling

$$g_i\,|G\rangle = |G\rangle \tag{5}$$

for all i, where

$$g_i = X_i \prod_{j=1}^{N} Z_j^{\Gamma_{i,j}} \tag{6}$$

and X_i is an operator acting with a generalised Pauli matrix X on the i'th subsystem (qudit) and with an identity operator $\mathbb{1}$ on every other qudit (similarly for Z_j). Note that only the number of edges $\Gamma_{i,j} \mod d$ matters in (6), so we are free to restrict our consideration to graphs for which $\Gamma_{i,j} \leqslant d-1$ holds for all i, j.

One of the most important properties of graph states is that some of them are equivalent to each other via local unitary operations. For example, we point out that the state $|C_4\rangle$ described in the main text (and repeated in Eq. (30) in Appendix II) is described by a set of stabilizers equivalent to $\mathbb{S} = \{ZZII, XXZI, IZXX, IIZZ\}$, whereas applying Eq. (6) would define the four-party linear graph state as stabilized by $\mathbb{S}' = \{XZII, ZXZI, IZXZ, IIZX\}$. However, $\mathbb{S}$ and $\mathbb{S}'$, and therefore their associated states, are equivalent up to the local unitary $U = HIIH$, where H is the Hadamard matrix. (Note that in the above, we have omitted the tensor product symbol $\otimes$ to lighten the notation.)

Interestingly, the equivalence under local unitaries can also be studied on the graphs themselves, i.e. for some graph transformations $G \to G'$ we know that there exists a local unitary operation $LU : |G\rangle \to |G'\rangle$ (see [1] for more details on this topic).

The first such operation is called *local complementation* (see Fig. 1a). This operation acts on a chosen vertex $n \in V$ such that the edges in the graph are modified according to the following formula

$$\Gamma'_{i,j} = \Gamma_{i,j} + \Gamma_{i,n}\Gamma_{j,n} \mod d\,, \tag{7}$$

where Γ' is the adjacency matrix of the graph after the transformation. Notice, that this changes only the neighbourhood of n (or more formally, the induced subgraph $G_S = (V_S = \mathcal{N}_n, E_S)$) while keeping the rest of the edges in the graph unchanged.

The second transformation of this type is *vertex multiplication* (see Fig. 1b), whose action on a vertex n is described by

$$\Gamma'_{i,n} = b\Gamma_{i,n} \mod d \quad \text{for all } i \in V\,, \qquad \Gamma'_{i,j} = \Gamma_{i,j} \quad \text{for all } i,j \neq n\,, \tag{8}$$

where $b \in \{1, \ldots, d-1\}$ can be chosen freely.

Using both of these transformations, one can find graph states that are related to each other via local unitaries. While this is a very difficult task in general, for some examples it is fairly straightforward. Let us consider one of the simplest examples relevant to our results: let us show that, given that d is prime, every graph state corresponding to a linear graph, where the number of vertices N can vary, is equivalent up to local unitaries to a graph state corresponding to a linear graph in which two consecutive vertices are connected by a single edge (called linear cluster state).

Without loss of generality, we can take that

$$\Gamma_{i,i+1} = \Gamma_{i+1,i} \neq 0 \mod d \quad \text{for all } i \in \{1, \ldots, N-1\}\,, \tag{9}$$

and $\Gamma_{i,j} = 0$ for all other i and j. Let us start by acting on vertex 2 with vertex multiplication such that

$$\Gamma'_{1,2} = b\Gamma_{1,2} = 1 \mod d. \tag{10}$$

Notice that we can always find such a b as long as d is prime. Next, we act on vertex 3 with vertex multiplication such that

$$\Gamma'_{2,3} = b\Gamma_{2,3} = 1 \mod d\,. \tag{11}$$

We can repeat this procedure until we get

$$\Gamma_{i,i+1} = 1 \quad \text{for all } i \in \{1, \ldots, N-2\}\,. \tag{12}$$

Lastly, to make $\Gamma'_{N-1,N} = 1$, we act with an appropriate vertex multiplication on the vertex N.

II. LOSR-GMNL DETECTION FOR A 4-QUBIT LINEAR CLUSTER STATE

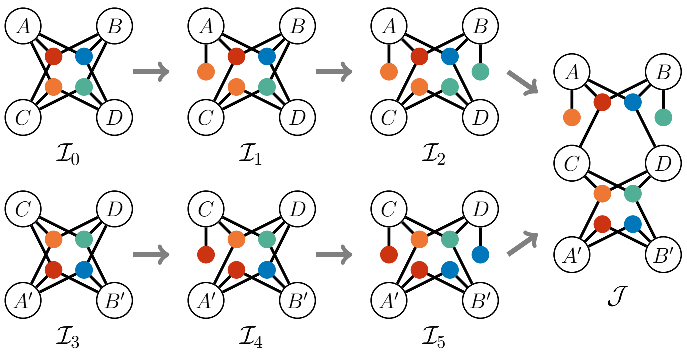

FIG. 2. Visual representation of the inflation used in the proof of Thm. 1. The colored disks represent general tripartite nonsignalling sources and the lettered disks are the parties. For clarity, we do not include in the figure classical randomness shared by all parties.

In this section, we derive a proof of LOSR-GMNL for an informative example of the 4-qubit linear cluster state, in order to show readers unfamiliar with the inflation technique how it works. Let us begin by recalling the monogamy relation from [2, Theorem 1],

$$I^{\tilde{C}D} + 2\langle \tilde{C}_0 E_0 \rangle \leqslant 4\,, \tag{13}$$

where $I^{\tilde{C}D}$ is the CHSH inequality [3]

$$I^{\tilde{C}D} = \langle \tilde{C}_0 D_0 \rangle + \langle \tilde{C}_0 D_1 \rangle + \langle \tilde{C}_1 D_0 \rangle - \langle \tilde{C}_1 D_1 \rangle \tag{14}$$

and $\tilde{C}_0, \tilde{C}_1$ are dichotomic observables with eigenvalues ± 1 representing measurements of the party $\tilde{C}$ (likewise for D_0, D_1, E_0). By substituting $\tilde{C}_0 = C_0$, $\tilde{C}_1 = A_0 C_1$, $E_0 = A'_1 B'_1$, where A_i, A'_i are observables belonging to parties A and A' respectively, we get the following inequality

$$I^{ACD} + 2\langle A'_1 B'_1 C_0 \rangle \leqslant 4\,, \tag{15}$$

where

$$I^{\tilde{C}D} = I^{ACD} = \langle C_0 D_0 \rangle + \langle C_0 D_1 \rangle + \langle A_0 C_1 D_0 \rangle - \langle A_0 C_1 D_1 \rangle\,. \tag{16}$$

Let us now consider the inflations presented in Fig. 2. From the assumption that parties in a network cannot communicate with each other classically, we can conclude that the monogamy relation (15) has to hold for any correlation originating from the inflation $\mathcal{J}$. The task now is to express the above inequality in terms of correlations originating from the original network, represented in Fig. 2 by $\mathcal{I}_0$.

Let us begin by considering the CHSH-like term I^{ACD} calculated for the correlations originating from the inflation $\mathcal{I}_1$. Since we assume that each copy of a party performs the same measurements and each copy of a source generates the same correlations, if the value of I^{ACD} is different for two inflations, it has to be due to the different network structures between two inflations. Moreover, notice that if the value I^{ACD} depended on which sources B is connected to, B could manipulate this value by choosing to perform measurements on some local correlations rather than correlations sent by the sources in the network. This way B could send information to the rest of the parties, and since we assume that parties cannot communicate with each other, we can conclude that the value of I^{ACD} can only depend on the structure of the subnetwork of parties A, C and D.

Consequently, since the subnetwork of parties A, C and D in $\mathcal{J}$ is equivalent to the same subnetwork in $\mathcal{I}_1$, it follows that the value of I^{ACD} is the same over correlations originating from $\mathcal{J}$ and $\mathcal{I}_1$:

$$I^{ACD}_{\mathcal{J}} = I^{ACD}_{\mathcal{I}_1}\,. \tag{17}$$

Furthermore, looking at each expected value in I^{ACD} individually, we can see that for $\langle C_0 D_0 \rangle$ and $\langle C_0 D_1 \rangle$ the only relevant subnetwork is actually the subnetwork of parties C and D, and since this subnetwork is equivalent in $\mathcal{J}$, $\mathcal{I}_1$ and $\mathcal{I}_0$ we have

$$\langle C_0 D_j \rangle_{\mathcal{J}} = \langle C_0 D_j \rangle_{\mathcal{I}_1} = \langle C_0 D_j \rangle_{\mathcal{I}_0} \tag{18}$$

for $j \in \{0, 1\}$. However, the same cannot be said for the expected values $\langle A_0 C_1 D_0 \rangle$ and $\langle A_0 C_1 D_1 \rangle$, since the subnetwork of parties A, C and D in I_0 is inequivalent to the same subnetwork in $\mathcal{I}_1$. Luckily, we can overcome this problem using an inequality from [4]

$$\langle A_0 C_1 D_0 \rangle_{\mathcal{I}_1} \geqslant \langle A_0 B_0 \rangle_{\mathcal{I}_1} + \langle B_0 C_1 D_0 \rangle_{\mathcal{I}_1} - 1\,, \tag{19}$$

(see Lemma 1 in Appendix III for a general formulation of this inequality).

Now, we can again argue that the subnetwork of parties A and B is equivalent in both $\mathcal{I}_1$ and $\mathcal{I}_0$, and that the subnetwork of parties B, C and D is also equivalent in these two inflations, hence

$$\langle A_0 B_0 \rangle_{\mathcal{I}_1} = \langle A_0 B_0 \rangle_{\mathcal{I}_0}\,, \qquad \langle B_0 C_1 D_0 \rangle_{\mathcal{I}_1} = \langle B_0 C_1 D_0 \rangle_{\mathcal{I}_0}\,. \tag{20}$$

We can follow the same argument to get

$$-\langle A_0 C_1 D_1 \rangle_{\mathcal{I}_1} \geqslant \langle A_0 B_0 \rangle_{\mathcal{I}_0} - \langle B_0 C_1 D_1 \rangle_{\mathcal{I}_1} - 1\,. \tag{21}$$

Putting it all together, we conclude that the value of $I^{ACD}_{\mathcal{J}}$ is bounded from below by

$$I^{ACD}_{\mathcal{J}} \geqslant I^{BCD}_{\mathcal{I}_0} + 2\langle A_0 B_0\rangle_{\mathcal{I}_0} - 2\,, \tag{22}$$

where

$$I^{BCD} = \langle C_0 D_0\rangle + \langle C_0 D_1\rangle + \langle B_0 C_1 D_0\rangle - \langle B_0 C_1 D_1\rangle\,. \tag{23}$$

Let us now look at the term $\langle C_0 A_1' B_1'\rangle$ in (15). Since the subnetwork of parties A', B' and C is the same in $\mathcal{J}$ as in $\mathcal{I}_4$, we have

$$\langle C_0 A_1' B_1'\rangle_{\mathcal{J}} = \langle C_0 A_1' B_1'\rangle_{\mathcal{I}_4}\,. \tag{24}$$

Next, we can use the inequality from [4] to get the following:

$$\langle C_0 A_1' B_1'\rangle_{\mathcal{I}_4} \geqslant \langle C_0 D_2\rangle_{\mathcal{I}_4} + \langle A_1' B_1' D_2\rangle_{\mathcal{I}_4} - 1\,. \tag{25}$$

Since the subnetworks of C and D and of A', B' and D are the same in $\mathcal{I}_3$ as in $\mathcal{I}_4$, we have

$$\langle C_0 D_2\rangle_{\mathcal{I}_4} = \langle C_0 D_2\rangle_{\mathcal{I}_3}\,, \qquad \langle A_1' B_1' D_2\rangle_{\mathcal{I}_4} = \langle A_1' B_1' D_2\rangle_{\mathcal{I}_3}\,. \tag{26}$$

Moreover, since $\mathcal{I}_3$ can be transformed into $\mathcal{I}_0$ by relabeling parties A' and B' to A and B, we can conclude that

$$\langle C_0 D_2\rangle_{\mathcal{I}_3} = \langle C_0 D_2\rangle_{\mathcal{I}_0}\,, \qquad \langle A_1' B_1' D_2\rangle_{\mathcal{I}_3} = \langle A_1 B_1 D_2\rangle_{\mathcal{I}_0}\,, \tag{27}$$

therefore

$$\langle C_0 A_1' B_1'\rangle_{\mathcal{J}} \geqslant \langle C_0 D_2\rangle_{\mathcal{I}_0} + \langle A_1 B_1 D_2\rangle_{\mathcal{I}_0} - 1\,. \tag{28}$$

Combining this with Ineq. (23) and (15), we get the final inequality, that has to be fulfilled for any correlations originating from $\mathcal{I}_0$,

$$I^{BCD}_{\mathcal{I}_0} + 2\langle A_0 B_0\rangle_{\mathcal{I}_0} + 2\langle C_0 D_2\rangle_{\mathcal{I}_0} + 2\langle A_1 B_1 D_2\rangle_{\mathcal{I}_0} \leqslant 8\,. \tag{29}$$

We can now use this inequality to show that the linear cluster state

$$|C_4\rangle = \frac{1}{2}\left(|0000\rangle + |0011\rangle + |1100\rangle - |1111\rangle\right) \tag{30}$$

is LOSR-GMNL. To this end, we take the observables

$$A_0 = B_0 = C_0 = D_2 = Z\,, \quad A_1 = B_1 = C_1 = X\,, \quad D_0 = \frac{1}{\sqrt{2}}(Z + X)\,, \quad D_1 = \frac{1}{\sqrt{2}}(Z - X)\,. \tag{31}$$

Computing the left-hand side of Ineq. (29) for those observables and a state $|C_4\rangle$ gives us the value $2\sqrt{2} + 6$ which violates the inequality. This implies that measurement statistics of $|C\rangle_4$ cannot be reproduced by the network $\mathcal{I}_0$, or, in other words, that $|C_4\rangle$ is LOSR-GMNL.

III. ALL CATERPILLAR STATES ARE LOSR-GMNL

In this section, we show that all graph states corresponding to caterpillar graphs (including, as a special case, all linear cluster states) are LOSR-GMNL. To this end, we first construct an inequality based on the monogamy relation from [2, Theorem 1] that has to be satisfied by any behavior originating from an N-partite network with $(N-1)$-partite sources of non-signalling correlations and an N-partite source of classical correlations. We then show that there exists a choice of measurements under which caterpillar states violate that inequality.

Let us begin by establishing the notation. An N-partite caterpillar state $|\ddagger_N\rangle$ is a graph state corresponding to a caterpillar graph with N vertices (see Appendix I for more details on graph states, including the definition of caterpillar graphs).

It will be convenient for the purposes of the proof to denote those vertices/parties not with a single number, but with a pair of numbers. To this end, we first need to find the longest induced line subgraph of the caterpillar graph. We denote the vertices belonging to this subgraph by $[i,0]$ for $i \in \{1,\ldots,L\}$, where L is the order (the number of vertices) of the line subgraph. We denote vertices that do not belong to said linear graph by $[i,j]$ for $j \geqslant 1$, where i is chosen such that $[i,j]$ is connected by an edge only to the vertex $[i,0]$. Notice that since vertices $\{[1,0],[2,0],\ldots,[L,0]\}$ form the longest induced line subgraph, there are no vertices $[i,j]$ such that $i=1,L$ and $j \geqslant 1$. See Fig. 3 for a visual example.

Under this notation, the state $|\ddagger_N\rangle$ is uniquely defined by the relations $g_{[i,j]}\,|\ddagger_N\rangle = |\ddagger_N\rangle$ for

$$\begin{aligned} g_{[1,0]} &= X_{[1,0]}Z_{[2,0]}\,, \\ g_{[i,0]} &= Z_{[i-1,0]}X_{[i,0]}Z_{[i+1,0]}\prod_{j=1}^{n_i} Z_{[i,j]} \quad \text{for } i \in \{2,\ldots,L-1\}\,, \\ g_{[i,j]} &= Z_{[i,0]}X_{[i,j]} \qquad \text{for } j \in \{1,\ldots,n_i\}\,, \\ g_{[L,0]} &= Z_{[L-1,0]}X_{[L,0]}\,, \end{aligned} \tag{32}$$

where $X_{[i,j]}, Z_{[i,j]}$ are Pauli operators acting on the $[i,j]$'th qubit (see Eq. (4) for $d=2$), $n_i = |\mathcal{N}_{[i,0]}| - 2$ and $\mathcal{N}_{[i,0]}$ is the neighbourhood of $[i,0]$ (see Appendix I for the definition).

The scenario considered in the proof is the following: an N-partite network $\mathcal{O}$ with N, $(N-1)$-partite sources of non-signalling correlations and an N-partite source of classical correlations. We label each party with the index $[i,j]$, as explained above, and we denote by $\tau_{[i,j]}$ a source distributing correlations to every party except party $[i,j]$. Moreover, we assume that the party $[L-1,0]$ can perform one of three measurements, $A_{[L-1,0];0}$, $A_{[L-1,0];1}$ or $A_{[L-1,0];2}$, while the rest of the parties $[i,j]$ can perform one of two measurements, $A_{[i,j];0}$ or $A_{[i,j];1}$.

The main method we use to construct the inequality capable of detecting LOSR-GMNL is the inflation technique. Given a network $\mathcal{O}$ described above, an inflation $\mathcal{I}$ of $\mathcal{O}$ is a network consisting of multiple copies of each party, multiple copies of each $(N-1)$-partite source of non-signalling correlations, and one global source of classical correlations. We assume that each copy of a given party is performing the same measurements as the original party in $\mathcal{O}$ and that each copy of a given source is distributing the same correlations as the original source $\tau_{[i,j]}$ in $\mathcal{O}$.

On top of the standard assumptions associated with the inflation technique, we also put additional assumptions on the inflations $\mathcal{I}$. First, we only consider inflations consisting of exactly two copies of each party $[i,j]$, which we denote by $[i,j]$ and $[i',j]$, and exactly two copies of each source $\tau_{[i,j]}$, which we denote by $\tau_{[i,j]}$ and $\tau_{[i',j]}$. Next, we require that party $[i,j]$ receives correlations from either $\tau_{[k,l]}$ or $\tau_{[k',l]}$ for all $[k,l] \neq [i,j]$. Together, these two assumptions imply that if party $[i,j]$ receives correlations from $\tau_{[k,l]}$ $(\tau_{[k',l]})$ then $[i',j]$ receives correlations from $\tau_{[k',l]}$ $(\tau_{[k,l]})$.

Let us consider a set of parties in $\mathcal{I}$ to which the source $\tau_{[i,j]}$ distributes correlations. Notice, that, using the above assumptions about $\mathcal{I}$, we can uniquely identify a source $\tau_{[i,j]}$ just by the set of non-primed parties that receive correlations from this source. Leveraging this fact, we introduce the notation $\tau_{[i,j]} = S$, where S is a set of non-primed parties that receive correlations from $\tau_{[i,j]}$. From the symmetry of $\mathcal{I}$ it follows that $\tau_{[i,j]} \cup \tau_{[i',j]} = P \setminus \{[i,j]\}$, where P is the set of all non-primed parties, which implies that the set $\{\tau_{[i,j]}\}_{[i,j]\in P}$ uniquely identifies $\mathcal{I}$.

To make the description of $\tau_{[i,j]}$ and other sets more concise, let us define the following sets:

$$\begin{aligned} (n,m)_1 &= \{n+i; i \in \{0,1,\ldots,m-n\}\}\,, \\ (n,m)_2 &= \{n+2i; i \in \{0,1,\ldots,(m-n)/2\}\}\,, \end{aligned} \tag{33}$$

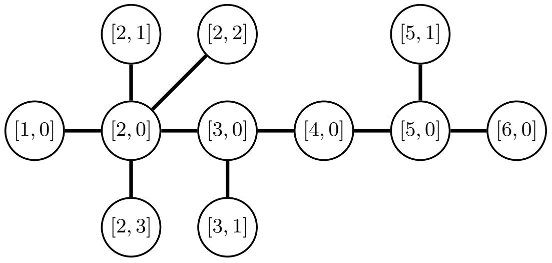

FIG. 3. Notation used for the vertices of a caterpillar graph. Note how the extremal vertices $[1,0]$ and $[6,0]$ have degree 1 by definition.

where we take $(n,m)_1 = \emptyset = (n,m)_2$ if $n > m$.

Lastly, we are going to use the following Lemma, which follows directly as a consequence of the results in [4].

Lemma 1. *Let M_1, M_2 and M_3 be mutually commuting observables with outcomes ± 1. Then the following inequality holds true:*

$$\langle M_1 M_2 \rangle \geqslant \langle M_1 M_3 \rangle + \langle M_2 M_3 \rangle - 1\,. \tag{34}$$

Please note, that this lemma does not require any assumptions about the type of probability distribution (like locality or non-signaling). It is a very general result that holds for any set of mutually commuting, dichotomic observables.

With this, we can formulate the main result of this section.

Theorem 4. *Let us consider an N-partite network with $(N-1)$-partite sources of non-signaling correlations and an N-partite source of classical correlations. Given that the party $[L-1,0]$ can perform one of three measurements while the rest of the parties have access to two measurements each and that $L \geqslant 3$, the resulting measurement probabilities are constrained by*

$$\begin{aligned} I_{L-2,L-1,L} + 2 \sum_{k\in(0,L-3)_1} \left(\left\langle A_{[k,0];0} A_{[k+1,0];1} A_{[k+2,0];0} \prod_{j=1}^{n_{k+1}} A_{[k+1,j];0} \right\rangle + 2 \sum_{l=1}^{n_{k+2}} \left\langle A_{[k+2,0];0} A_{[k+2,l];1} \right\rangle \right) \\ + 2 \left\langle A_{[L-1,0];0} A_{[L,0];1} \right\rangle \leqslant 2(2N-L)\,, \end{aligned} \tag{35}$$

where

$$\begin{aligned} I_{L-2,L-1,L} = \left\langle A_{[L-1,0];1} A_{[L,0];1} \right\rangle + \left\langle A_{[L-2,0];0} A_{[L-1,0];1} A_{[L,0];0} \prod_{j=1}^{n_{L-1}} A_{[L-1,j];0} \right\rangle \\ - \left\langle A_{[L-1,0];2} A_{[L,0];1} \right\rangle + \left\langle A_{[L-2,0];0} A_{[L-1,0];2} A_{[L,0];0} \prod_{j=1}^{n_{L-1}} A_{[L-1,j];0} \right\rangle . \end{aligned} \tag{36}$$

We divide the following proof into two parts. First, we give a complete proof for even L, then we give a proof for odd L where we skip some steps if the derivation is the same for even and odd L.

Proof. Part 1: even L

Let us begin by considering a network inflation $\mathcal{I}_0$ defined as

$$\mathcal{I}_0: \quad \tau_{[i,j]} = \begin{cases} [(1,i-2)_2 \cup (i,L)_1, \cdot] \setminus \{[i,j]\} & \text{for odd } i \text{ and all } j\,, \\ [(2,i-2)_2 \cup (i,L)_1, \cdot] \setminus \{[i,j]\} & \text{for even } i \text{ and all } j\,, \end{cases} \tag{37}$$

where for an arbitrary set S we have $[S,\cdot] = \{[l,r]\}_{l\in S, r\in(0,n_l)_1}$. Using [2, Theorem 1], we can write a monogamy relation that holds true in $\mathcal{I}_0$

$$I_{L-1,L;t}\big|_{\mathbf{a}_e=(-1)^t} + 2\left\langle A_{[1',0];0} \prod_{i\in(2,L)_2} A_{[i,0];1} \prod_{j=1}^{n_i} A_{[i,j];0} \right\rangle \Big|_{\mathbf{a}_e=(-1)^t} \leqslant 4\,, \tag{38}$$

where

$$\mathbf{a}_e = \left(\prod_{i\in(1,L-3)_2} a_{[i,0];1} \prod_{j=1}^{n_i} a_{[i,j];0} \right) \prod_{j=1}^{n_{L-1}} a_{[L-1,j];0}\,, \tag{39}$$

where $a_{[i,j];k}$ is a measurement result of $A_{[i,j];k}$, and

$$I_{L-1,L;t} = \langle A_{[L-1,0];1} A_{[L,0];1} \rangle + (-1)^t \langle A_{[L-1,0];1} A_{[L,0];0} \rangle - \langle A_{[L-1,0];2} A_{[L,0];1} \rangle + (-1)^t \langle A_{[L-1,0];2} A_{[L,0];0} \rangle\,. \tag{40}$$

We multiply both sides of (38) by $P(\mathbf{a}_e = (-1)^t)$ and we sum over $t \in \{0,1\}$

$$I_{L-1,L;\mathrm{av}} + 2\left\langle A_{[1',0];0} \prod_{i\in(2,L)_2} A_{[i,0];1} \prod_{j=1}^{n_i} A_{[i,j];0} \right\rangle \leqslant 4\,, \tag{41}$$

where

$$I_{L-1,L;\mathrm{av}} = \sum_{t=0}^{1} P(\mathbf{a}_e = (-1)^t) I_{L-1,L;t}|_{\mathbf{a}_e=(-1)^t} \,. \tag{42}$$

Our goal is to formulate an inequality similar to Ineq. (41) but for the original network $\mathcal{O}$ rather than an inflation $\mathcal{I}_0$. To this end, we have to bound the value of both terms on the left-hand side of (41) by some other expected values calculated over correlations from $\mathcal{O}$.

First, let us focus on $I_{L-1,L;\mathrm{av}}$. Using the fact that

$$\sum_{t=0}^{1} P(\mathbf{a}_e = (-1)^t) \left\langle A_{[L-1,0];j} A_{[L,0];1} \right\rangle |_{\mathbf{a}_e=(-1)^t} = \left\langle A_{[L-1,0];j} A_{[L,0];1} \right\rangle \tag{43}$$

for $j \in \{1, 2\}$, we can rewrite $I_{L-1,L;\mathrm{av}}$ as

$$\begin{aligned} I_{L-1,L;\mathrm{av}} = &\sum_{t=0}^{1} (-1)^t P(\mathbf{a}_e = (-1)^t) \left\langle A_{[L-1,0];1} A_{[L,0];0} \right\rangle |_{\mathbf{a}_e=(-1)^t} + \sum_{t=0}^{1} (-1)^t P(\mathbf{a}_e = (-1)^t) \left\langle A_{[L-1,0];2} A_{[L,0];0} \right\rangle |_{\mathbf{a}_e=(-1)^t} \\ &+ \left\langle A_{[L-1,0];1} A_{[L,0];1} \right\rangle - \left\langle A_{[L-1,0];2} A_{[L,0];1} \right\rangle \,. \end{aligned} \tag{44}$$

Let us focus on the first sum. We can expand the expected value in terms of probabilities which yields

$$\begin{aligned} \sum_{t=0}^{1} (-1)^t P(\mathbf{a}_e = (-1)^t) \left\langle A_{[L-1,0];1} A_{[L,0];0} \right\rangle |_{\mathbf{a}_e=(-1)^t} &= \sum_{t,k=0}^{1} (-1)^{t+k} P(\mathbf{a}_e = (-1)^t) P(a_{[L-1,0];1} a_{[L,0];0} = (-1)^k | \mathbf{a}_e = (-1)^t) \\ &= \sum_{t,k=0}^{1} (-1)^{t+k} P(a_{[L-1,0];1} a_{[L,0];0} = (-1)^k, \mathbf{a}_e = (-1)^t) \\ &= \sum_{l=0}^{1} (-1)^l P(\mathbf{a}_e a_{[L-1,0];1} a_{[L,0];0} = (-1)^l) = \left\langle \mathbf{A}_e A_{[L-1,0];1} A_{[L,0];0} \right\rangle \,, \end{aligned} \tag{45}$$

where

$$\mathbf{A}_e = \left(\prod_{i \in (1,L-3)_2} A_{[i,0];1} \prod_{j=1}^{n_i} A_{[i,j];0} \right) \prod_{j=1}^{n_{L-1}} A_{[L-1,j];0} \,. \tag{46}$$

Applying the same technique for the second sum in (44) allows us to conclude that

$$I_{L-1,L;\mathrm{av}} = \left\langle A_{[L-1,0];1} A_{[L,0];1} \right\rangle + \left\langle \mathbf{A}_e A_{[L-1,0];1} A_{[L,0];0} \right\rangle - \left\langle A_{[L-1,0];2} A_{[L,0];1} \right\rangle + \left\langle \mathbf{A}_e A_{[L-1,0];2} A_{[L,0];0} \right\rangle \,. \tag{47}$$

Notice that this expression depends on measurements on parties $[(1, L-1)_2, \cdot]$ and on a party $[L, 0]$. Since a subnetwork consisting of those parties in $\mathcal{I}_0$ has the same structure as a subnetwork consisting of the same parties in $\mathcal{I}_1$, defined as

$$\mathcal{I}_1 : \quad \tau_{[i,j]} = \begin{cases} [(1, L)_1, \cdot] \setminus \{[i, j]\} & \text{for odd } i \text{ and all } j \,, \\ [(i, L)_1, \cdot] \setminus \{[i, j]\} & \text{for even } i \text{ and all } j \,, \end{cases} \tag{48}$$

we conclude that the value of (47) calculated over correlations from $\mathcal{I}_0$ is equal to the value of (47) calculated over correlations from $\mathcal{I}_1$.

While we cannot relate the value of (47) calculated over $\mathcal{I}_1$ directly to the value of (47) calculated over $\mathcal{O}$, since the structure of a relevant subnetwork is different for $\mathcal{I}_1$ and $\mathcal{O}$, we can achieve it by performing an intermediate step.

The plan is to bound the value of (47) from below by a function of other expected values in $\mathcal{I}_1$, which then can be related to the same expected values in $\mathcal{O}$. To this end, we make use of Lemma 1 to formulate the following inequality:

$$\begin{aligned}
I_{L-1,L;\text{av}} &= \langle A_{[L-1,0];1}A_{[L,0];1}\rangle + \langle \mathbf{A}_e A_{[L-1,0];1}A_{[L,0];0}\rangle - \langle A_{[L-1,0];2}A_{[L,0];1}\rangle + \langle \mathbf{A}_e A_{[L-1,0];2}A_{[L,0];0}\rangle \\
&\geqslant \langle A_{[L-1,0];1}A_{[L,0];1}\rangle + \langle T_0\mathbf{A}_e A_{[L-1,0];1}A_{[L,0];0}\rangle + \langle T_0\rangle - 1 \\
&\quad - \langle A_{[L-1,0];2}A_{[L,0];1}\rangle + \langle T_0\mathbf{A}_e A_{[L-1,0];2}A_{[L,0];0}\rangle + \langle T_0\rangle - 1 \\
&\geqslant \langle A_{[L-1,0];1}A_{[L,0];1}\rangle + \left\langle A_{[L-2,0];0}A_{[L-1,0];1}A_{[L,0];0}\prod_{j=1}^{n_{L-1}} A_{[L-1,j];0}\right\rangle \\
&\quad - \langle A_{[L-1,0];2}A_{[L,0];1}\rangle + \left\langle A_{[L-2,0];0}A_{[L-1,0];2}A_{[L,0];0}\prod_{j=1}^{n_{L-1}} A_{[L-1,j];0}\right\rangle + 2\sum_{k\in(0,L-4)_2}(\langle T_k\rangle - 1) \\
&= I_{L-2,L-1,L} + 2\sum_{k\in(0,L-4)_2}\langle T_k\rangle - L + 2\,,
\end{aligned} \tag{49}$$

where $I_{L-2,L-1,L}$ is defined in (36),

$$T_k = A_{[k,0];0}A_{[k+1,0];1}A_{[k+2,0];0}\prod_{j=1}^{n_{k+1}} A_{[k+1,j];0}\,, \tag{50}$$

for any $k \in (0, L-1)_1$ and $A_{[0,0];0} = A_{[L+1,0];0} = \mathbb{1}$, and we used the fact that

$$\mathbf{A}_e = A_{[L-2,0];0}\prod_{j=1}^{n_{L-1}} A_{[L-1,j],0}\prod_{k\in(0,L-4)_2} T_k\,. \tag{51}$$

Let us now consider the original network $\mathcal{O}$

$$\mathcal{O}: \quad \tau_{[i,j]} = [(1,L)_1,\cdot]\setminus\{[i,j]\}\,. \tag{52}$$

Notice that the subnetwork consisting of parties $[L-2,0]$, $[L-1,\cdot]$ and $[L,0]$ has the same structure in both $\mathcal{I}_1$ and $\mathcal{O}$, which implies that the value of $I_{L-2,L-1,L}$ is the same in both of these networks. As for the terms $\langle T_k\rangle$, they cannot be related to $\mathcal{O}$ directly, as the subnetworks on which the T_k terms act nontrivially are different in $\mathcal{I}_1$ and in $\mathcal{O}$. However, this problem can be averted by using a set of inflation tailored individually for each T_k.

Let us consider the following set of inflations

$$\mathcal{J}_k^m: \quad \tau_{[i,j]} = \begin{cases} [(i,L)_1,\cdot]\setminus\{[i,j]\} & \text{for } i = k+2 \text{ and all } j \geqslant m+1\,,\\ [(1,L)_1,\cdot]\setminus\{[i,j]\} & \text{else}\,. \end{cases} \tag{53}$$

Based on the equivalence of the relevant subnetworks, we have that, for $k \in (0, L-4)_2$, expected values $\langle T_k\rangle$ calculated over a state from $\mathcal{I}_1$ and from $\mathcal{J}_k^0$ are equal. We can now use Lemma 1 to get the following inequality:

$$\langle T_k\rangle \geqslant \left\langle A_{[k,0];0}A_{[k+1,0];1}A_{[k+2,1];1}\prod_{j=1}^{n_{k+1}} A_{[k+1,j];0}\right\rangle + \langle A_{[k+2,0];0}A_{[k+2,1];1}\rangle - 1\,. \tag{54}$$

Since the subnetworks $\{[k+2,0],[k+2,1]\}$ in $\mathcal{J}_k^0$ and $\mathcal{O}$ are equivalent, we get that the second expected value on the right-hand side of the inequality calculated over correlations from $\mathcal{J}_k^0$ and $\mathcal{O}$ yields the same value. As for the first expected value on the right-hand side, we can conclude that the expected value calculated over correlations from $\mathcal{J}_k^0$ equals the same expected value calculated over correlations from $\mathcal{J}_k^1$.

We can again use Lemma 1 to bound this expected value by

$$\left\langle A_{[k,0];0}A_{[k+1,0];1}A_{[k+2,1];1}\prod_{j=1}^{n_{k+1}} A_{[k+1,j];0}\right\rangle \geqslant \left\langle A_{[k,0];0}A_{[k+1,0];1}A_{[k+2,2];1}\prod_{j=1}^{n_{k+1}} A_{[k+1,j];0}\right\rangle + \langle A_{[k+2,1];1}A_{[k+2,2];1}\rangle - 1\,. \tag{55}$$

Again, we can relate the second expected value on the right to the same expected value calculated over correlations from $\mathcal{O}$, while relating the first expected value to the same expected value calculated over correlations from the inflation $\mathcal{J}_k^2$.

We repeat this procedure until we get to the following inequality,

$$\begin{aligned}\left\langle A_{[k,0];0}A_{[k+1,0];1}A_{[k+2,n_{k+2}-1];1}\prod_{j=1}^{n_{k+1}}A_{[k+1,j];0}\right\rangle \geqslant \left\langle A_{[k,0];0}A_{[k+1,0];1}A_{[k+2,n_{k+2}];1}\prod_{j=1}^{n_{k+1}}A_{[k+1,j];0}\right\rangle \\ +\left\langle A_{[k+2,n_{k+2}-1];1}A_{[k+2,n_{k+2}];1}\right\rangle - 1\,,\end{aligned} \tag{56}$$

where the expected values are calculated over correlations from $\mathcal{J}_k^{n_{k+2}-1}$. We can now use the same technique again to conclude that both expected values on the right side of the inequality are equal to the same expected values but calculated over correlations from $\mathcal{O}$.

In order for the final inequality to be well defined for any value of n_{k+2} we have to use Lemma 1 twice more:

$$\begin{aligned}\left\langle A_{[k,0];0}A_{[k+1,0];1}A_{[k+2,n_{k+2}];1}\prod_{j=1}^{n_{k+1}}A_{[k+1,j];0}\right\rangle \geqslant \left\langle A_{[k,0];0}A_{[k+1,0];1}A_{[k+2,0];0}\prod_{j=1}^{n_{k+1}}A_{[k+1,j];0}\right\rangle \\ +\left\langle A_{[k+2,0];0}A_{[k+2,n_{k+2}];1}\right\rangle - 1\,,\end{aligned} \tag{57}$$

and

$$\left\langle A_{[k+2,l];1}A_{[k+2,l+1];1}\right\rangle \geqslant \left\langle A_{[k+2,l];1}A_{[k+2,0];0}\right\rangle + \left\langle A_{[k+2,l+1];1}A_{[k+2,0];0}\right\rangle - 1 \tag{58}$$

for all $l \in (1, n_{k+2}-1)_1$.

The above method can be applied for all $k \in (0, L-4)_2$ meaning that every expected value $\langle T_k\rangle$ calculated over correlations from $\mathcal{I}_1$ can be bounded from below by expected values calculated over correlations from $\mathcal{O}$. Putting it all together we get

$$\begin{aligned}(I_{L-1,L,\mathrm{av}})_{\mathcal{I}_0} \geqslant I_{L-2,L-1,L} + 2\sum_{k\in(0,L-4)_2}\Bigg(\left\langle A_{[k,0];0}A_{[k+1,0];1}A_{[k+2,0];0}\prod_{j=1}^{n_{k+1}}A_{[k+1,j];0}\right\rangle \\ + 2\sum_{l=1}^{n_{k+2}}\left\langle A_{[k+2,l];1}A_{[k+2,0];0}\right\rangle - 2n_{k+2}\Bigg) - L + 2\,,\end{aligned} \tag{59}$$

where by writing $(I_{L-1,L,\mathrm{av}})_{\mathcal{I}_0}$, we emphasize that the left-hand side of the inequality is calculated on correlations from $\mathcal{I}_0$ while the right-hand side is calculated on correlations from $\mathcal{O}$.

We now come back to Ineq. (41) and we focus on the term $\langle A_{[1',0];0}\prod_{i\in(2,L)_2}A_{[i,0];1}\prod_{j=1}^{n_i}A_{[i,j];0}\rangle$ in the inflation $\mathcal{I}_0$. Let us consider the following inflation:

$$\mathcal{I}_2: \quad \tau_{[i,j]} = \begin{cases}(\{[1,0]\}\cup[(i,L)_1,\cdot])\setminus\{[i,j]\} & \text{for odd } i \text{ and all } j\,,\\ [(2,N)_1,\cdot]\setminus\{[i,j]\} & \text{for even } i \text{ and all } j\,.\end{cases} \tag{60}$$

Due to the equivalence of the relevant subnetworks in $\mathcal{I}_0$ and $\mathcal{I}_2$, we have that the expected value $\langle A_{[1',0];0}\prod_{i\in(2,L)_2}A_{[i,0];1}\prod_{j=1}^{n_i}A_{[i,j]}$ calculated over correlations from $\mathcal{I}_0$ is equal to the same expected value calculated for the correlations from $\mathcal{I}_2$.

Let us now consider

$$\mathcal{I}_3: \quad \tau_{[i,j]} = \begin{cases}[(i,L)_1,\cdot]\setminus\{[i,j]\} & \text{for odd } i \text{ and all } j\,,\\ [(1,N)_1,\cdot]\setminus\{[i,j]\} & \text{for even } i \text{ and all } j\,.\end{cases} \tag{61}$$

This inflation is derived from $\mathcal{I}_2$ by simply swapping the labels of parties 1 and 1′. We therefore have that $\langle A_{[1',0];0}\prod_{i\in(2,L)_2}A_{[i,0];1}\prod_{j=1}^{n_i}A_{[i,j];0}\rangle$ calculated over correlations from $\mathcal{I}_2$ equals $\langle A_{[1,0];0}\prod_{i\in(2,L)_2}A_{[i,0];1}\prod_{j=1}^{n_i}A_{[i,j];0}\rangle$ (notice the lack of a prime in the first observable) calculated over correlations from $\mathcal{I}_3$.

We can bound this expected value using Lemma 1 and the fact that

$$A_{[1,0];0}\prod_{i\in(2,L)_2}A_{[i,0];1}\prod_{j=1}^{n_i}A_{[i,j];0} = \prod_{k\in(1,L-1)_2}T_k\,, \tag{62}$$

which gives us

$$\left\langle A_{[1,0];0} \prod_{i\in(2,L)_2} A_{[i,0];1} \prod_{j=1}^{n_i} A_{[i,j];0} \right\rangle \geqslant \left\langle A_{[1,0];0} \prod_{i\in(2,L-2)_2} A_{[i,0];1} \prod_{j=1}^{n_i} A_{[i,j];0} A_{[L-1,0];0} \right\rangle + \langle T_{L-1}\rangle - 1$$
$$\geqslant \sum_{k\in(1,L-1)_2} \langle T_k\rangle - \frac{L}{2} + 1 \,. \tag{63}$$

In order to bound the expected values $\langle T_k\rangle$ by correlations from $\mathcal{O}$, we once again make use of the inflations $\mathcal{J}_k^m$ in the exact same way as before, which results in the following inequality,

$$\left\langle A_{[1',0];0} \prod_{i\in(2,L)_2} A_{[i,0];1} \prod_{j=1}^{n_i} A_{[i,j],0} \right\rangle_{\mathcal{I}_0} \geqslant \sum_{k\in(1,L-1)_2} \Bigg(\left\langle A_{[k,0];0} A_{[k+1,0];1} A_{[k+2,0];0} \prod_{j=1}^{n_{k+1}} A_{[k+1,j];0} \right\rangle$$
$$+ 2 \sum_{l=1}^{n_{k+2}} \left\langle A_{[k+2,l];1} A_{[k+2,0];0} \right\rangle - 2n_{k+2} \Bigg) - \frac{L}{2} + 1 \,, \tag{64}$$

where expected values on the right-hand side of the inequality are calculated over correlations from $\mathcal{O}$.

We can finally combine Ineq.s (41), (59) and (64) into a single inequality

$$I_{L-2,L-1,L} + 2 \sum_{k\in(0,L-3)_1} \Bigg(\left\langle A_{[k,0];0} A_{[k+1,0];1} A_{[k+2,0];0} \prod_{j=1}^{n_{k+1}} A_{[k+1,j];0} \right\rangle + 2 \sum_{l=1}^{n_{k+2}} \left\langle A_{[k+2,0];0} A_{[k+2,l];1} \right\rangle \Bigg)$$
$$+ 2 \left\langle A_{[L-1,0];0} A_{[L,0];1} \right\rangle \leqslant 2(2N-L) \,. \tag{65}$$

Part 2: odd L

Let us begin by considering network inflation $\mathcal{I}_0$ defined in Eq. (37). Using [2, Theorem 1], we can write a monogamy relation that holds true in $\mathcal{I}_0$,

$$I_{L-1,L;t}|_{\mathbf{a}_o=(-1)^t} + 2 \left\langle \prod_{i\in(1,L)_2} A_{[i,0];1} \prod_{j=1}^{n_i} A_{[i,j];0} \right\rangle |_{\mathbf{a}_o=(-1)^t} \leqslant 4 \,, \tag{66}$$

where

$$\mathbf{a}_o = a_{[1',0];0} \left(\prod_{i\in(2,L-3)_2} a_{[i,0];1} \prod_{j=1}^{n_i} a_{[i,j];0} \right) \prod_{j=1}^{n_{L-1}} a_{[L-1,j];0} \,. \tag{67}$$

We multiply both sides of (66) by $P(\mathbf{a}_o = (-1)^t)$ and we sum over $t \in \{0,1\}$

$$I_{L-1,L;\mathrm{av}} + 2 \left\langle \prod_{i\in(1,L)_2} A_{[i,0];1} \prod_{j=1}^{n_i} A_{[i,j];0} \right\rangle \leqslant 4 \,, \tag{68}$$

where

$$I_{L-1,L;\mathrm{av}} = \sum_{t=0}^{1} P(\mathbf{a}_o = (-1)^t) I_{L-1,L;t}|_{\mathbf{a}_o=(-1)^t} \,. \tag{69}$$

Using the same technique as in the first part of the proof, we get

$$I_{L-1,L;\mathrm{av}} = \left\langle A_{[L-1,0];1} A_{[L,0];1} \right\rangle + \left\langle \mathbf{A}_o A_{[L-1,0];1} A_{[L,0];0} \right\rangle - \left\langle A_{[L-1,0];2} A_{[L,0];1} \right\rangle + \left\langle \mathbf{A}_o A_{[L-1,0];2} A_{[L,0];0} \right\rangle \,, \tag{70}$$

where

$$\mathbf{A}_o = A_{[1',0];0} \left(\prod_{i\in(2,L-3)_2} A_{[i,0];1} \prod_{j=1}^{n_i} A_{[i,j];0} \right) \prod_{j=1}^{n_{L-1}} A_{[L-1,j];0} \,. \tag{71}$$

Our goal is to formulate an inequality similar to Ineq. (68) but over correlations from the original network $\mathcal{O}$ rather than an inflation $\mathcal{I}_0$.

Let us first focus on $I_{L-1,L;\mathrm{av}}$. Notice that this expression depends only on measurements on parties $[(2,L-1)_2,\cdot]$, $[1',0]$, $[L,0]$. Since a subnetwork consisting of those parties in $\mathcal{I}_0$ is equivalent to a subnetwork consisting of the same parties in $\mathcal{I}_2$, defined by Eq. (60), we conclude that the value of $I_{L-1,L;\mathrm{av}}$ calculated over correlations from $\mathcal{I}_0$ is equal to the value of $I_{L-1,L;\mathrm{av}}$ calculated over correlations from $\mathcal{I}_2$.

Moreover, $I_{L-1,L;\mathrm{av}}$ calculated over correlations from $\mathcal{I}_2$ is equal to

$$I_{L-1,L;\tilde{\mathrm{av}}} = \left\langle A_{[L-1,0];1}A_{[L,0];1}\right\rangle + \left\langle \tilde{\mathbf{A}}_o A_{[L-1,0];1}A_{[L,0];0}\right\rangle - \left\langle A_{[L-1,0];2}A_{[L,0];1}\right\rangle + \left\langle \tilde{\mathbf{A}}_o A_{[L-1,0];2}A_{[L,0];0}\right\rangle \tag{72}$$

calculated over correlations from $\mathcal{I}_3$, where

$$\tilde{\mathbf{A}}_o = A_{[1,0];0}\left(\prod_{i\in(2,L-3)_2} A_{[i,0];1}\prod_{j=1}^{n_i} A_{[i,j];0}\right)\prod_{j=1}^{n_{L-1}} A_{[L-1,j];0}\,. \tag{73}$$

As was the case for even L, here we can bound the value of $I_{L-1,L;\tilde{\mathrm{av}}}$ from below by

$$I_{L-1,L;\tilde{\mathrm{av}}} \geqslant I_{L-2,L-1,L} + 2\sum_{k\in(1,L-4)_2}\langle T_k\rangle - L + 3\,, \tag{74}$$

where $I_{N-2,N-1,N}$ is defined in (36).

Let us now consider the original network $\mathcal{O}$ (52). Since the relevant subnetwork for $I_{L-2,L-1,L}$ is equivalent in $\mathcal{I}_3$ and $\mathcal{O}$, the value of $I_{L-2,L-1,L}$ calculated over correlations from $\mathcal{I}_3$ and $\mathcal{O}$ is the same. As for $\langle T_k\rangle$, these expected values can be bounded from below by utilizing inflations $\mathcal{J}_k^m$ (53) for $m\in(0,n_{k+2}-1)$, which at the end gives us

$$\begin{aligned}(I_{L-1,L,\mathrm{av}})_{\mathcal{I}_0} \geqslant I_{L-2,L-1,L} + 2\sum_{k\in(1,L-4)_2}\Bigg(&\left\langle A_{[k,0];0}A_{[k+1,0];1}A_{[k+2,n_{k+2}];1}\prod_{j=1}^{n_{k+1}} A_{[k+1,j];0}\right\rangle \\ &+2\sum_{l=1}^{n_{k+2}}\left\langle A_{[k+2,l];1}A_{[k+2,0];0}\right\rangle - 2n_{k+2}\Bigg) - L + 3\,,\end{aligned} \tag{75}$$

where the left-hand side is calculated over correlations from $\mathcal{O}$.

We now come back to Ineq. (68) and we focus on the term $\langle\prod_{i\in(1,L)_2} A_{[i,0];1}\prod_{j=1}^{n_i} A_{[i,j];0}\rangle$. Notice that it depends only on parties $[(1,L)_2,\cdot]$. Since $\mathcal{I}_0$ (37) and $\mathcal{I}_1$ (48) differ only by connections to parties $[(2,L-1)_2,\cdot]$, we can conclude that

$$\left\langle\prod_{i\in(1,L)_2} A_{[i,0];1}\prod_{j=1}^{n_i} A_{[i,j];0}\right\rangle_{\mathcal{I}_0} = \left\langle\prod_{i\in(1,L)_2} A_{[i,0];1}\prod_{j=1}^{n_i} A_{[i,j];0}\right\rangle_{\mathcal{I}_1}\,. \tag{76}$$

We then use Lemma 1 to bound this expected value from below

$$\left\langle\prod_{i\in(1,L)_2} A_{[i,0];1}\prod_{j=1}^{n_i} A_{[i,j];0}\right\rangle \geqslant \sum_{k\in(0,L-1)_2}\langle T_k\rangle - \frac{L-1}{2}\,. \tag{77}$$

Next, each $\langle T_k\rangle$ can itself be bounded from below using the inflations $\mathcal{J}_k^m$ (53), which in the end results in

$$\begin{aligned}\left\langle\prod_{i\in(1,L)_2} A_{[i,0];1}\prod_{j=1}^{n_i} A_{[i,j];0}\right\rangle_{\mathcal{I}_0} \geqslant \sum_{k\in(0,L-1)_2}\Bigg(&\left\langle A_{[k,0];0}A_{[k+1,0];1}A_{[k+2,n_{k+2}];1}\prod_{j=1}^{n_{k+1}} A_{[k+1,j];0}\right\rangle \\ &+2\sum_{l=1}^{n_{k+2}}\left\langle A_{[k+2,l];1}A_{[k+2,0];0}\right\rangle - 2n_{k+2}\Bigg) - \frac{L-1}{2}\,,\end{aligned} \tag{78}$$

where the right-hand side is evaluated over correlations from $\mathcal{O}$.

Putting (75) and (78) together, we get the following inequality fulfilled by correlations from $\mathcal{O}$

$$I_{L-2,L-1,L} + 2 \sum_{k\in(0,L-3)_1} \left(\left\langle A_{[k,0];0} A_{[k+1,0];1} A_{[k+2,0];0} \prod_{j=1}^{n_{k+1}} A_{[k+1,j];0} \right\rangle + 2 \sum_{l=1}^{n_{k+2}} \left\langle A_{[k+2,0];0} A_{[k+2,l];1} \right\rangle \right) + 2 \left\langle A_{[L-1,0];0} A_{[L,0];1} \right\rangle \leqslant 2(2N-L)\,. \tag{79}$$

□

After deriving an inequality detecting LOSR-GMNL, we can now show that the caterpillar state $|\ddagger_N\rangle$, as well as any state ρ sufficiently close to it, is LOSR-GMNL.

Corollary 1. *Let us consider a state $\rho_{\ddagger_N}(\eta)$ defined as*

$$\rho_{\ddagger_N}(\eta) = \eta\, |\ddagger_N\rangle\langle\ddagger_N| + \frac{1-\eta}{2^N}\mathbb{1}\,. \tag{80}$$

*Given that $L \geqslant 3$, $\rho_{\ddagger_N}(\eta)$ is LOSR-*GMNL *for*

$$\eta > \frac{2N-L}{2N-L+\sqrt{2}-1}\,. \tag{81}$$

Proof. We take the measurements as follows:

$$\begin{aligned} A_{[i,j];0} = Z\,, \quad A_{[i,j];1} = X \qquad &\text{for } [i,j] \in [(1,L)_1, \cdot] \setminus \{[L-1,0]\}\,, \\ A_{[L-1,0];0} = Z\,, \quad A_{[L-1,0];1} = \frac{1}{\sqrt{2}}(X+Z)\,, \quad &A_{[L-1,0];2} = \frac{1}{\sqrt{2}}(X-Z)\,. \end{aligned} \tag{82}$$

and then, using the stabilizing operators (32) of a caterpillar graph state, we calculate each term in Ineq. (35) for the state $\rho_{\ddagger_N}(\eta)$:

$$I_{L-2,L-1,L} = 2\sqrt{2}\eta\,, \quad \langle\cdot\rangle = \eta\,, \tag{83}$$

where by $\langle\cdot\rangle$ we mean that it holds true for all expected values in Ineq. (35). Substituting this into (35) yields Ineq. (81). □

IV. LINEAR CLUSTER STATES ARE LOSR-GMNL FOR ANY PRIME LOCAL DIMENSION

In this section, we prove that a linear cluster state for any prime local dimension is LOSR-GMNL. We achieve this by utilizing the inflation technique to show that the assumption that the linear cluster state is not LOSR-GMNL violates a monogamy relation from [2]. Most of the details concerning the considered scenario (the inflation technique, etc.) are the same here as in Appendix III, so we will not repeat them here. There are only three substantial changes that need to be highlighted: different party labels, different quantum states under examination, and a generalized monogamy relation suitable for subsystems with local dimension higher than two.

First, here we label all of the parties simply using a set of numbers $\{1,\ldots,N\}$. Second, here we consider the linear cluster state $\left|C_N^d\right\rangle$ a state of prime local dimension d which is stabilized by

$$\begin{aligned} g_1 &= X_1 Z_2\,, \\ g_i &= Z_{i-1} X_i Z_{i+1} \quad \text{for } i \in (2, N-1)_1\,, \\ g_N &= Z_{N-1} X_N\,, \end{aligned} \tag{84}$$

where X and Z are defined in (4) (for an overview of graph states see Appendix I).

Third, the aforementioned monogamy relation [2, Theorem 1] is given by

$$I_{m,n} + d(1 - P(a_{n;i} = a_{l;k})) \geqslant d-1\,, \tag{85}$$

where $a_{l;k} \in \{\omega^j\}_{j=0}^{d-1}$ denotes the measurement outcome of measurement k of any party $l \neq m, n$ and

$$I_{m,n} = \sum_{k=0}^{d-1} \sum_{i=1}^{2} k\Big(P(a_{m;i} = \omega^k a_{n;i}) + P(a_{n;i} = \omega^{k+\delta_{i,1}} a_{m;i+1})\Big) \geqslant d-1 \tag{86}$$

is the Collins, Gisin, Linden, Massar, Popescu (CGLMP) inequality [5], where $a_{m;3} \equiv a_{m;1}$. Measurements that lead to a violation of CGLMP inequality $I_{m,n}$ by a maximally entangled state $|\phi^+\rangle := \frac{1}{\sqrt{d}} \sum_j |jj\rangle$ [5] can be presented as the following observables

$$A_{m;j} = U_{\alpha_j} X^\dagger U_{\alpha_j}^\dagger , \qquad A_{n;j} = U_{\beta_j} X U_{\beta_j}^\dagger , \tag{87}$$

where $U_\chi = \sum_j \omega^{j\chi} |j\rangle\langle j|$ for any χ, and $\alpha_1 = 0$, $\alpha_2 = 1/2$, $\beta_1 = 1/4$, $\beta_2 = -1/4$.

For the purposes of our proof, we need to slightly adjust these observables. First, we need the first measurement of party n to be equal to X. To this end, we make use of the property $V^* \otimes V |\phi^+\rangle = |\phi^+\rangle$ that holds for all unitary matrices V. Taking $V = U_{\beta_1}$ gives us a set of observables that also violate CGLMP with $|\phi^+\rangle$. In terms of Eq. (87) the new observables are described by the following set of coefficients $\alpha_1 = 1/4$, $\alpha_2 = 3/4$, $\beta_1 = 0$, $\beta_2 = -1/2$.

The last adjustment that we need to make is that we need a violation of CGLMP (86) for the state $|C_2^d\rangle$ rather than $|\phi^+\rangle$. We use the relation $|C_2^d\rangle = F \otimes \mathbb{1} |\phi^+\rangle$, where F is the discrete Fourier transform matrix

$$F = \sum_{i,j=0}^{d-1} \omega^{ij} |i\rangle\langle j| , \tag{88}$$

to find that the state $|C_2^d\rangle$ violates CGLMP inequality (86) with observables

$$A_{m;j} = F U_{\alpha_j} X^\dagger U_{\alpha_j}^\dagger F^\dagger , \qquad A_{n;j} = U_{\beta_j} X U_{\beta_j}^\dagger , \tag{89}$$

with coefficients $\alpha_1 = 1/4$, $\alpha_2 = 3/4$, $\beta_1 = 0$ and $\beta_2 = -1/2$.

With that, we can proceed to the main result of this section.

Theorem 5. *A linear cluster state $|C_N^d\rangle$ is LOSR-*GMNL *for all prime d and $N \geqslant 3$.*

As was the case in Appendix III, here we also split the proof into two parts. We first show a complete proof for even N, after which we show a proof for odd N in which we omit derivations common for the cases of even and odd N.

Proof. Part 1: even N

We begin by describing correlations that can be produced by the linear cluster $|C_N^d\rangle$, and we then prove by contradiction that these correlations cannot be produced in the network.

From Eq. (84), it follows that, from the projection of $|C_N^d\rangle$ onto the state $|0\rangle_{N-2}$, we get a state $|\psi\rangle$ fulfilling

$$|\psi\rangle = \eta\, |0\rangle_{N-2}\langle 0|\ |C_N^d\rangle = \eta\, |0\rangle_{N-2}\langle 0|\ g_i\, |C_N^d\rangle = \eta g_i\, |0\rangle_{N-2}\langle 0|\ |C_N^d\rangle = g_i\, |\psi\rangle \tag{90}$$

for all $i \neq N-2$, where η is the normalization constant. From this relation it follows that $|\psi\rangle$ is stabilized by

$$\begin{aligned} g_1 &= X_1 Z_2 , \qquad g_i = Z_{i-1} X_i Z_{i+1} \quad \text{for } i \in (2, N-4)_1 , \\ g_{N-3} &= Z_{N-4} X_{N-3} , \qquad g_{N-2} = Z_{N-2} , \\ g_{N-1} &= X_{N-1} Z_N , \qquad g_N = Z_{N-1} X_N . \end{aligned} \tag{91}$$

Therefore, we have that

$$|\psi\rangle = |C_{N-3}\rangle_{1,\ldots,N-3} \otimes |0\rangle_{N-2} \otimes |C_2^d\rangle . \tag{92}$$

Let us choose the following observables:

$$A_{i;j} = X^j Z^{1-j} \quad \text{for } i \in (1, N-2)_1 , \qquad A_{N-1;j} = F U_{\beta_j}^\dagger X^\dagger U_{\beta_j} F^\dagger , \qquad A_{N-1;0} = Z , \qquad A_{N;j} = U_{\alpha_j}^\dagger X U_{\alpha_j} . \tag{93}$$

Under this choice of observables, we have

$$I_{N-1,N}|_{a_{N-2;0}=1} < d-1\,, \tag{94}$$

and, from the stabilizing operators (84) of $\left|C_N^d\right\rangle$, we can infer the expected values

$$\langle A_{i;0}A_{i+1;1}A_{i+2;0}\rangle = 1 \qquad \forall \qquad i \in (0, N-3)_1\,, \tag{95}$$

which imply that

$$P(a_{i;0}a_{i+1;1}a_{i+2;0} = 1) = 1 \qquad \forall \qquad i \in (0, N-3)_1\,, \tag{96}$$

where $a_{i;j}$ is the measurement result of measurement $A_{i;j}$ and we take $a_{0;0} := 1$. We also have $\langle A_{N-1;0}A_{N;1}\rangle = 1$, and therefore

$$P(a_{N-1;0}a_{N;1} = 1) = 1\,. \tag{97}$$

We will now show that the (94), (96) and (97) cannot be satisfied simultaneously by any correlations originating from a network $\mathcal{O}$ consisting of N parties connected with $(N-1)$-partite sources of non-signalling correlations and an N-partite source of shared randomness. We will achieve this by assuming the contrary, i.e. that (94), (96) and (97) hold true for some correlation from $\mathcal{O}$, which will lead us to a contradiction.

More specifically, we consider a network inflation

$$\mathcal{I}_0: \quad \tau_i = \begin{cases} (1, i-2)_2 \cup (i, L)_1 \setminus \{i\} & \text{for odd } i\,,\\ (2, i-2)_2 \cup (i, L)_1 \setminus \{i\} & \text{for even } i\,, \end{cases} \tag{98}$$

in which we will show that the above set of assumptions leads to the violation of the following monogamy relation (85):

$$I_{N-1,N}|_{a_{1;1}^{\xi_1}a_{3;1}^{\xi_3}\ldots a_{N-3;1}^{\xi_{N-3}}=1} + d(1 - P(a_{1';0}^{\chi_1}a_{2;1}^{\chi_2}a_{4;1}^{\chi_4}\ldots, a_{N;1}^{\chi_N} = 1|a_{1;1}^{\xi_1}a_{3;1}^{\xi_3}\ldots a_{N-3;1}^{\xi_{N-3}} = 1)) \geqslant d-1\,, \tag{99}$$

where $I_{N-1,N}$ is the CGLMP inequality defined in (86),

$$\chi_n = \begin{cases} 1 & \text{for } n = 1,2 \mod 4\,,\\ d-1 & \text{for } n = 0,3 \mod 4\,, \end{cases} \tag{100}$$

and $\xi_j = -\chi_j\chi_{N-3}^{-1} \mod d$, with χ_{N-3}^{-1} the multiplicative inverse of χ_{N-3} in $\mathbb{Z}_d$ (since d is prime, this inverse is guaranteed to exist).

We begin by considering what we can infer about the value of $I_{N-1,N}|_{a_{1;1}^{\xi_1}a_{3;1}^{\xi_3}\ldots a_{N-3;1}^{\xi_{N-3}}=1}$ from the assumptions (94), (96) and (97). To this end, let us consider the original network $\mathcal{O}$ and its inflation $\mathcal{I}_1$

$$\mathcal{I}_1: \quad \tau_i = \begin{cases} (1, L)_1 \setminus \{i\} & \text{for odd } i\,,\\ (i, L)_1 \setminus \{i\} & \text{for even } i\,. \end{cases} \tag{101}$$

Notice that the subnetworks consisting of three consecutive parties $i, i+1, i+2$ for $i \in (0, N-2)_2$ (we ignore party 0) have the same structure in $\mathcal{O}$ and in $\mathcal{I}_1$ (101). Therefore, by the assumption that (94), (96) and (97) are true in $\mathcal{O}$, we conclude that conditions

$$I_{N-1,N}|_{a_{N-2;0}=0} < d-1\,, \qquad a_{i;0}a_{i+1;1}a_{i+2;0} = 1 \qquad \forall\ i \in (0, N-2)_2 \tag{102}$$

hold in $\mathcal{I}_1$. It follows then, that

$$(a_{1;1}a_{2;0})^{\chi_1}(a_{2;0}a_{3;1}a_{4;0})^{\chi_3}\ldots(a_{N-4;0}a_{N-3;1}a_{N-2;0})^{\chi_{N-3}} = 1 \tag{103}$$

where χ_i are defined in Eq. (100), and the equality follows from the fact that each term in parentheses equals 1. Using Eq. (100), we can then conclude that

$$(a_{1;1}a_{2;0})^{\chi_1}(a_{2;0}a_{3;1}a_{4;0})^{\chi_3}\ldots(a_{N-4;0}a_{N-3;1}a_{N-2;0})^{\chi_{N-3}} = a_{1;1}^{\chi_1}a_{3;1}^{\chi_3}\ldots a_{N-3;1}^{\chi_{N-3}}a_{N-2;0}^{\chi_{N-3}} = 1\,, \tag{104}$$

which in turn implies:

$$a_{N-2;0} = \prod_{i\in(1,N-3)_2} a_{i;1}^{\xi_i}\,, \tag{105}$$

where, as a reminder, $\xi_i = -\chi_i\chi_{N-3}^{-1} \mod d$. Substituting for $a_{N-2;0}$ in $I_{N-1,N}|_{a_{N-2;0}=1}$ gives us

$$I_{N-1,N}|_{a_{1;1}^{\xi_1}a_{3;1}^{\xi_3}\ldots a_{N-3;1}^{\xi_{N-3}}=1} < d-1\,. \tag{106}$$

Notice that this expression depends only on the measurement results of odd-numbered parties and the party N. The subnetwork consisting of the relevant parties is equivalent in $\mathcal{I}_1$ and $\mathcal{I}_0$ (98), so we conclude that (106) holds true for $\mathcal{I}_0$ if (94), (96) and (97) hold in the original network.

Coming back to the monogamy relation (99), we can now calculate the value of the term

$$P(a_{1';0}^{\chi_1}a_{2;1}^{\chi_2}a_{4;1}^{\chi_4}\ldots,a_{N;1}^{\chi_N}=1|a_{1;1}^{\xi_1}a_{3;1}^{\xi_3}\ldots a_{N-3;1}^{\xi_{N-3}}=1)\,. \tag{107}$$

We start again from $\mathcal{O}$ and use the consequences of our assumption that (94), (96) and (97) can be fulfilled by a correlation originating from $\mathcal{O}$. In particular, this means that

$$a_{i;0}a_{i+1;1}a_{i+2;0}=1\,,\quad \text{for all } i\in(1,\ldots,N-3)_2, \qquad a_{N-1;0}a_{N;1}=1 \tag{108}$$

hold true in $\mathcal{O}$. Since the subnetwork containing any three parties $i, i+1, i+2$ for $i \in (1,\ldots,N-1)_2$ (we ignore the party $N+1$) has the same structure in $\mathcal{O}$ as in $\mathcal{I}_3$, where

$$\mathcal{I}_3: \quad \tau_i = \begin{cases} (i,L)_1\setminus\{i\} & \text{for odd } i\,,\\ (1,N)_1\setminus\{i\} & \text{for even } i\,,\end{cases} \tag{109}$$

we conclude that (108) also holds true in $\mathcal{I}_3$. Furthermore, since $\mathcal{I}_2$

$$\mathcal{I}_2: \quad \tau_i = \begin{cases} \{1\}\cup(i,L)_1\setminus\{i\} & \text{for odd } i\,,\\ (2,N)_1\setminus\{i\} & \text{for even } i\,,\end{cases} \tag{110}$$

and $\mathcal{I}_3$ can be transformed into each other by swapping parties 1 and $1'$, we find that

$$a_{1';0}a_{2;1}a_{3;0}=1\,,\quad a_{i;0}a_{i+1;1}a_{i+2;0}=1 \quad \text{for all } i\in(3,\ldots,N-3)_2\,, \qquad a_{N-1;0}a_{N;1}=1 \tag{111}$$

hold true in $\mathcal{I}_2$.

Taking a product of all of the relations in $\mathcal{I}_2$ with appropriate powers gives us

$$(a_{1';0}a_{2;1}a_{3;0})^{\chi_2}(a_{3;0}a_{4;1}a_{5;0})^{\chi_4}\ldots(a_{N-1;0}a_{N;1})^{\chi_N} = a_{1';0}^{\chi_1}a_{2;1}^{\chi_2}a_{4;1}^{\chi_4}\ldots,a_{N;1}^{\chi_N}=1\,, \tag{112}$$

where we use the fact that $\chi_1=\chi_2$. This relation depends only on measurements by even-numbered parties plus the party $1'$. Since the appropriate subnetworks in $\mathcal{I}_2$ and $\mathcal{I}_0$ are equivalent, this implies that Eq. (112) holds true for $\mathcal{I}_0$. Therefore,

$$P(a_{1';0}^{\chi_1}a_{2;1}^{\chi_2}a_{4;1}^{\chi_4}\ldots,a_{N;1}^{\chi_N}=1)=1 \tag{113}$$

for $\mathcal{I}_0$. Since this probability is strictly 1, it follows that

$$P(a_{1';0}^{\chi_1}a_{2;1}^{\chi_2}a_{4;1}^{\chi_4}\ldots,a_{N;1}^{\chi_N}=1|a_{1;1}^{\xi_1}a_{3;1}^{\xi_3}\ldots a_{N-3;1}^{\xi_{N-3}}=1)=1\,. \tag{114}$$

Combining this result with (106) results in the following bound,

$$I_{N-1,N}|_{a_{1;1}^{\xi_1}a_{3;1}^{\xi_3}\ldots a_{N-3;1}^{\xi_{N-3}}=1} + d(1-P(a_{1';1}^{\chi_1}a_{2;0}^{\chi_2}a_{4;0}^{\chi_4}\ldots,a_{N;0}^{\chi_N}=1|a_{1;1}^{\xi_1}a_{3;1}^{\xi_3}\ldots a_{N-3;1}^{\xi_{N-3}}=1)) < d-1\,, \tag{115}$$

which violates monogamy (99).

Part 2: odd N

Since this part of the proof is very similar to the case of even N, we only showcase a general proof technique while skipping details explained in the previous part.

Our goal is to perform a proof by contradiction: we show that the assumption that (94), (96) and (97) is fulfilled by a correlation originating from $\mathcal{O}$ results in a violation of the following monogamy relation:

$$I_{N-1,N}|_{a_{1';0}^{\xi_1}a_{2;1}^{\xi_2}a_{4;1}^{\xi_4}\dots a_{N-3;1}^{\xi_{N-3}}=1} + d(1 - P(a_{1;1}^{\chi_1}a_{3;1}^{\chi_3}\dots, a_{N;1}^{\chi_N} = 1|a_{1';0}^{\xi_1}a_{2;1}^{\xi_2}a_{4;1}^{\xi_4}\dots a_{N-3;1}^{\xi_{N-3}} = 1)) \geqslant d-1\,, \tag{116}$$

where $I_{N-1,N}$ is the CGLMP inequality (86), χ_j is defined in Eq. (100), and $\xi_j = -\chi_j\chi_{N-3}^{-1}$.

Looking at the subnetworks consisting of parties $i, i+1, i+2$ for $i \in (1, N-2)_2$ in $\mathcal{O}$ and $\mathcal{I}_3$ (109), we can conclude that, by our assumption, the relations

$$I_{N-1,N}|_{a_{N-2;0}=1} < d-1\,, \qquad a_{i;0}a_{i+1;1}a_{i+2;0} = 1 \quad \text{for } i \in (1, N-4)_2\,, \tag{117}$$

hold true in $\mathcal{I}_3$. Swapping parties 1 and $1'$ gives us the equivalent relations in $\mathcal{I}_2$ (110), which we can use to derive the following:

$$a_{1';0}^{\chi_1}a_{2;1}^{\chi_2}a_{4;1}^{\chi_4}\dots a_{N-3;1}^{\chi_{N-3}}a_{N-2;0}^{\chi_{N-3}} = 1\,. \tag{118}$$

We use this to derive a formula for $a_{N-2;0}$, which allows us to show that

$$I_{N-1,N}|_{a_{1';0}^{\xi_1}a_{2;1}^{\xi_2}a_{4;1}^{\xi_4}\dots a_{N-3;1}^{\xi_{N-3}}=1} < d-1 \tag{119}$$

holds true in $\mathcal{I}_2$. Next, analysing the appropriate subnetworks of $\mathcal{I}_2$ and $\mathcal{I}_0$ (98), one can conclude that the above relation also holds in $\mathcal{I}_0$.

On the other hand, from our assumptions and from the equivalence of the appropriate subnetworks, we can conclude that

$$a_{i;0}a_{i+1;1}a_{i+2;0} = 1 \quad \text{for all } i \in (0, N-3)_2\,, \qquad a_{N-1;0}a_{N;1} = 1 \tag{120}$$

holds true in $\mathcal{I}_1$ (101). Taking a product of these expressions with appropriate powers gives us

$$a_{1;1}^{\chi_1}a_{3;1}^{\chi_3}\dots, a_{N;1}^{\chi_N} = 1\,. \tag{121}$$

This can be shown to hold true in $\mathcal{I}_0$ by the equivalence of subnetworks containing only odd-numbered, non-primed parties in inflations $\mathcal{I}_1$ and $\mathcal{I}_0$. We therefore have that

$$P(a_{1;1}^{\chi_1}a_{3;1}^{\chi_3}\dots, a_{N;1}^{\chi_N} = 1|a_{1';0}^{\xi_1}a_{2;1}^{\xi_2}a_{4;1}^{\xi_4}\dots a_{N-3;1}^{\xi_{N-3}} = 1) = 1\,. \tag{122}$$

Combining the above with (119), we get

$$I_{N-1,N}|_{a_{1';0}^{\xi_1}a_{2;1}^{\xi_2}a_{4;1}^{\xi_4}\dots a_{N-3;1}^{\xi_{N-3}}=1} + d(1 - P(a_{1;1}^{\chi_1}a_{3;1}^{\chi_3}\dots, a_{N;1}^{\chi_N} = 1|a_{1';0}^{\xi_1}a_{2;1}^{\xi_2}a_{4;1}^{\xi_4}\dots a_{N-3;1}^{\xi_{N-3}} = 1)) < d-1\,, \tag{123}$$

which violates Ineq. (116). □

V. THE GHZ STATE IS LOSR-GMNL FOR ANY LOCAL DIMENSION

In this section, we generalize the results from [6] to show that a generalized Greenberger-Horne-Zeilinger (GHZ) [7] state

$$\left|\mathrm{GHZ}_N^d\right\rangle = \sum_{j=0}^{d-1} |j\rangle^{\otimes N} \tag{124}$$

is LOSR-GMNL for any d. Crucially, this state is stabilized by the following operators

$$\begin{aligned} g_1 &= X_1X_2\dots X_N\,, \\ g_j &= Z_{j-1}Z_j^{-1} \quad \text{for } j \in (2, N)_1\,, \end{aligned} \tag{125}$$

where X and Z are defined in Eq. (4).

As was the case in Appendix IV, here we also make use of the CGLMP inequality [5] defined in (86). For more details on this inequality, see Appendix IV. We also use the same assumption and notation for inflations as established in Appendix III.

Theorem 6. *A GHZ state* $\left|\mathrm{GHZ}_N^d\right\rangle$ *is LOSR-*GMNL *for any* d *and* $N \geqslant 3$.

Proof. We prove the theorem by contradiction. To this end, let us take the observables as follows:

$$A_{1;k} = F^\dagger U_{\alpha_k} X^\dagger U_{\alpha_k}^\dagger F\,, \qquad A_{2;k} = F U_{\beta_k} X U_{\beta_k}^\dagger F^\dagger\,, \qquad A_{2;0} = Z\,, \qquad A_{i;j} = Z^{1-j}X^j \quad , \tag{126}$$

where $k \in \{1,2\}$, $j \in \{0,1\}$, $i \in (3,N)_1$, F is the discrete Fourier transform matrix and $U_\chi = \sum_j \omega^{j\chi} |j\rangle\langle j|$ for any χ, with $\alpha_1 = 0$, $\alpha_2 = 1/2$, $\beta_1 = 1/4$, $\beta_2 = -1/4$. Note that $A_{1;1} = F^\dagger X^\dagger F = Z$. Let us denote by $|\phi\rangle_{3,\ldots,N}$ the state on parties $3,\ldots,N$ after the measurement that results in $a_{3;1}a_{4;1}\ldots a_{N;1} = 1$. From (125), it follows that the global state $|\psi\rangle$ after the measurement fulfills

$$|\psi\rangle = \eta\,|\phi\rangle_{3,\ldots,N}\,\langle\phi|\left|\mathrm{GHZ}_N^d\right\rangle = \eta\,|\phi\rangle_{3,\ldots,N}\,\langle\phi|\,g_1\left|\mathrm{GHZ}_N^d\right\rangle = \eta X_1X_2\,|\phi\rangle_{3,\ldots,N}\,\langle\phi|\left|\mathrm{GHZ}_N^d\right\rangle = X_1X_2\,|\psi\rangle\;, \tag{127}$$

where η is a normalization constant. Similarly, using g_2, one can show that $|\psi\rangle = Z_1Z_2^{-1}|\psi\rangle$, therefore

$$|\psi\rangle = \left|\phi^+\right\rangle_{1,2}|\phi\rangle_{3,\ldots,N}\;, \tag{128}$$

where $|\phi^+\rangle_{1,2} = 1/\sqrt{d}\sum_{j=0}^{d-1}|jj\rangle$. We can then use the fact that $F\otimes F^\dagger|\phi^+\rangle = |\phi^+\rangle$ to conclude that, using observables $A_{1;1}, A_{1;2}, A_{2;1}, A_{2;2}$ from Eq. (126), we get

$$I_{1,2}|_{a_{3;1}a_{4;1}\ldots a_{N;1}=1} < d-1\,. \tag{129}$$

Moreover, from (125) we have

$$\langle A_{1;1}A_{2;0}^{-1}\rangle = 1\,, \quad \langle A_{i;0}A_{i+1;0}^{-1}\rangle = 1 \quad \text{for } i \in (2,N-1)_1\,. \tag{130}$$

Note that measuring the observable Z^{-1} is equivalent to measuring Z but assigning to each outcome the multiplicative inverse of the corresponding outcome of Z, so from (130) we have

$$a_{1;1}a_{2;0}^{-1} = 1\,, \quad a_{i;0}a_{i+1;0}^{-1} = 1 \quad \text{for } i \in (2,N-1)_1\,, \tag{131}$$

and therefore

$$a_{1;1} = a_{2;0}\,, \quad a_{i;0} = a_{i+1;0} \quad \text{for } i \in (2,N-1)_1\,. \tag{132}$$

We will now show that the correlations (129) and (132) cannot be produced simultaneously by a network $\mathcal{O}$ consisting of N parties connected by $(N-1)$-partite sources of non-signalling correlations and an N-partite source of shared randomness.

Let us consider the following inflation of $\mathcal{O}$

$$\mathcal{I}_0: \quad \tau_i = (1,N)_1 \setminus \{i\}\,. \tag{133}$$

First, we use the results of [2] to establish the following monogamy relation,

$$I_{1,2}|_{a_{3;1}a_{4;1}\ldots a_{N;1}=1} + d(1 - P(a_{1;1} = a_{N';0}|a_{3;1}a_{4;1}\ldots a_{N;1} = 1)) \geqslant d-1\,, \tag{134}$$

which holds in $\mathcal{I}_0$. The CGLMP term does not depend on any primed parties, and, since $\mathcal{I}_0$ consists of two disconnected copies of $\mathcal{O}$, we have that its value is exactly the same in $\mathcal{I}_0$ as in $\mathcal{O}$. If we assume then that (129) can be achieved in $\mathcal{O}$, it must also be achieved in $\mathcal{I}_0$. Consider now the intermediary inflation

$$\mathcal{I}_1: \quad \tau_i = (i+1,N)_1\,. \tag{135}$$

Notice that any subnetwork consisting of two parties $j, j+1$ for all $j \in (1,N-1)_1$ has the same structure in $\mathcal{O}$ and in $\mathcal{I}_1$. Hence, if the correlations (132) hold in $\mathcal{O}$, they will also hold in $\mathcal{I}_1$. What's more, one can combine all of the equalities in (132) to find

$$a_{1;1} = a_{N;0}\,. \tag{136}$$

In $\mathcal{I}_1$, parties 1 and N do not share any common source, which is also true for parties 1 and N' in $\mathcal{I}_0$. Therefore, in $\mathcal{I}_0$ we have

$$a_{1;1} = a_{N';0}\,, \tag{137}$$

so, trivially,

$$P(a_{1;1} = a_{N';0}|a_{3;1}a_{4;1}\dots a_{N;1} = 1) = P(a_{1;1} = a_{N';0}) = 1\,. \tag{138}$$

Combined with (129), this implies that

$$I_{1,2}|_{a_{3;1}a_{4;1}\dots a_{N;1}=1} + d(1 - P(a_{1;1} = a_{N';0}|a_{3;1}a_{4;1}\dots a_{N;1} = 1)) < d - 1\,. \tag{139}$$

This contradicts the monogamy relation (134) and proves that $\left|\mathrm{GHZ}_N^d\right\rangle$ is LOSR-GMNL. □

VI. LONC-GMNL SETTING

A. $|\mathrm{GHZ}_n\rangle$ is maximally LONC-GMNL on a directed path

We prove Theorem 3 which appears in the main text.

Theorem 7 (Full statement of Theorem 3 from the main text.)**.** *In the LONC-*GMNL *model, the following inequality holds if we allow $t < n-1$ rounds of synchronous communication along an oriented path, where the n parties are named $A^{(1)} \to \cdots \to A^{(n)}$.*

$$\langle A_1^{(1)} \cdots A_1^{(n-1)} A_1^{(n)}\rangle + \langle A_1^{(1)} \cdots A_1^{(n-1)} A_2^{(n)}\rangle + \langle A_0^{(1)} A_1^{(n)}\rangle - \langle A_0^{(1)} A_2^{(n)}\rangle + 2\langle A_0^{(1)} A_0^{(n)}\rangle \leq 4\,. \tag{140}$$

It is violated by the quantum state $|\mathrm{GHZ}_n\rangle$ when measuring the observables Z for $A_0^{(1)}, A_0^{(n)}$; X for $A_1^{(1)}, \dots, A_1^{(n-1)}$; $\frac{X+Z}{\sqrt{2}}$ for $A_1^{(n)}$; and $\frac{X-Z}{\sqrt{2}}$ for $A_2^{(n)}$.

Proof. The quantum violation is straightforward. We prove the inequality by invoking the monogamy of bipartite CHSH non-signaling correlations [2]. We first remark that $t < n-1$ implies that no causal resources, except for the starting classical shared randomness, can be shared by the extremal parties $A^{(1)}$ and $A^{(n)}$ in an oriented path. An external party $\tilde{A}^{(n)}$ having access only to the shared randomness can thus achieve the same correlation $2\langle A_0^{(1)} A_0^{(n)}\rangle = 2\langle A_0^{(1)} \tilde{A}_0^{(n)}\rangle$. Then, we observe that the first four terms of the left-hand side of Eq. (140) are reducible to the CHSH inequality by regrouping the parties into two groups: $(A^{(1)} \dots A^{(n-1)})$ and $A^{(n)}$. We note that the oriented path structure prevents signalling between those two groups. More precisely, the inputs $a \in \{0,1\}$ given to $A^{(1)}$ starts too far to reach $A^{(n)}$ in $t < n-1$ steps and a thus cannot effect the marginal probability distribution $P(A_b^{(n)}|a) = P(A_b^{(n)})$; and the other input, let it be $b \in \{0,1,2\}$, given to $A^{(n)}$ cannot effect the joint marginal probability distribution $P(A_a^{(1)} \dots A_1^{(n-1)}|b) = P(A_a^{(1)} \dots A_1^{(n-1)})$ because the signalling is only left to right. We conclude by applying the monogamy inequality for bipartite non-signaling correlations that are given in [2, Equation 2]. □

B. Linear cluster states are (only) LONC-GMNL_2 on a directed path

Here we show a way of generating a linear cluster state in 2 rounds of communication on an N-partite line network in which communication is allowed only in one direction. For simplicity, we take that the first party can send information to the second, the second to the third, etc. Each party $i \neq 1, N$ has access to two qubits, which we denote $(i,1)$ and $(i,2)$, each of them initially in a state $|+\rangle$. As for the first and last party, the first one starts with three qubits in a state $|+\rangle$, which we denote by $(1,1)$, $(1,2)$ and $(0,1)$, while the party N starts with no qubits. We thus start with a graph state corresponding to an edgeless graph with $2N-1$ vertices. In what follows, we give an algorithm to generate a linear cluster state in one-way line networks; however, we will describe the operations in terms of graph transformations. Afterward, we comment on how these transformations relate to the operations performed on qubits.

1. Each party i connects vertices $(i,1)$ and $(i,2)$ with an edge. The first party additionally connects $(0,1)$ and $(1,1)$.
2. Each party $i \neq N$ sends vertex (qubit) $(i,2)$ to the party $i+1$.
3. Each party $i \neq 1, N$ connects $(i,1)$ and $(i-1,2)$ with an edge.

4. We perform local complementation on vertices $(i, 2)$ which in turn connects vertices $(i, 1)$ and $(i+1, 1)$ for all $i \neq 0$.

5. Each party $i \neq 1, N$ disconnects vertices $(i, 1)$ and $(i-1, 2)$.

6. Party i sends vertex $(i, 1)$ to the party $i+1$.

7. Each party $i \neq 1$ disconnects vertices $(i-1, 1)$ and $(i-1, 2)$. The remaining graph is a linear graph of qubits (i,1) for all $i \in \{0, \ldots, N-1\}$ and isolated vertices $(i, 2)$ for all $i \in \{1, \ldots, N\}$.

The procedure is illustrated for $N = 4$ in Fig. 4. The operation of connecting and disconnecting vertices corresponds to the action of $C_Z = \text{diag}(1, 1, 1, -1)$ on the corresponding qubits. In the above algorithm, this operation is always performed locally, i.e. on qubits that a given party has access to. As for the local complementation, it is known that this operation corresponds to action with local unitaries on individual qubits (see Appendix I for more details).

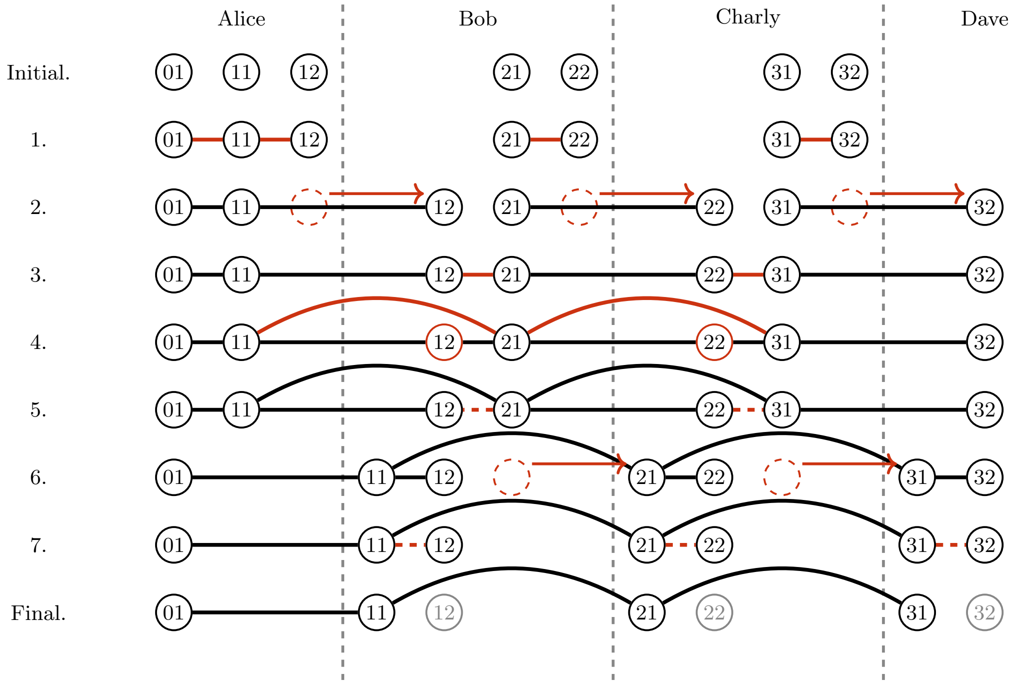

FIG. 4. An illustration for $N = 4$ of our quantum algorithm to prepare a linear cluster state with local operations and two rounds of synchronous communication on a directed path.

[1] M. Bahramgiri and S. Beigi, Graph states under the action of local clifford group in non-binary case (2007), arXiv:quant-ph/0610267.
[2] R. Augusiak, M. Demianowicz, M. Pawłowski, J. Tura, and A. Acín, Elemental and tight monogamy relations in nonsignaling theories, Physical Review A **90**, 10.1103/physreva.90.052323 (2014).
[3] J. F. Clauser, M. A. Horne, A. Shimony, and R. A. Holt, Proposed experiment to test local hidden-variable theories, Phys. Rev. Lett. **23**, 880 (1969).
[4] M. Navascué s, E. Wolfe, D. Rosset, and A. Pozas-Kerstjens, Genuine network multipartite entanglement, Physical Review Letters **125**, 10.1103/physrevlett.125.240505 (2020).
[5] D. Collins, N. Gisin, N. Linden, S. Massar, and S. Popescu, Bell inequalities for arbitrarily high-dimensional systems, Phys. Rev. Lett. **88**, 040404 (2002).
[6] X. Coiteux-Roy, E. Wolfe, and M.-O. Renou, Any physical theory of nature must be boundlessly multipartite nonlocal, Phys. Rev. A **104**, 052207 (2021).
[7] D. M. Greenberger, M. A. Horne, and A. Zeilinger, Going beyond Bell's theorem, in *Bell's Theorem, Quantum Theory and Conceptions of the Universe*, edited by M. Kafatos (Springer Netherlands, Dordrecht, 1989) pp. 69–72.
